# Overexpression screen of chromosome 21 genes reveals modulators of Sonic hedgehog signaling relevant to Down syndrome

**DOI:** 10.1242/dmm.049712

**Published:** 2023-04-13

**Authors:** Anna J. Moyer, Fabian-Xosé Fernandez, Yicong Li, Donna K. Klinedinst, Liliana D. Florea, Yasuhiro Kazuki, Mitsuo Oshimura, Roger H. Reeves

**Affiliations:** ^1^ Department of Genetic Medicine, Johns Hopkins University School of Medicine, Baltimore, MD 21205, USA; ^2^ Department of Physiology, Johns Hopkins University School of Medicine, Baltimore, MD 21205, USA; ^3^ Department of Neurobiology, University of Alabama at Birmingham, Birmingham, AL 35233, USA; ^4^ Department of Psychology, University of Arizona, Tucson, AZ 85724, USA; ^5^ Department of Neurology, University of Arizona, Tucson, AZ 85724, USA; ^6^ BIO5 and McKnight Brain Research Institutes, Tucson, AZ 85721, USA; ^7^ Division of Genome and Cellular Functions, Department of Molecular and Cellular Biology, School of Life Science, Faculty of Medicine and Chromosome Engineering Research Center, Tottori University, 86 Nishi-cho, Yonago, Tottori 683-8503, Japan; ^8^ Chromosome Engineering Research Center, Tottori University, Yonago, Tottori 683-8503, Japan; ^9^ Trans Chromosomics, Inc., 86 Nishi-cho, Yonago, Tottori 683-8503, Japan

**Keywords:** Down syndrome, Sonic hedgehog, Aneuploidy, Gene dosage effects, Genetic screen, Trisomy 21

## Abstract

Trisomy 21 and mutations in the Sonic hedgehog (SHH) signaling pathway cause overlapping and pleiotropic phenotypes including cerebellar hypoplasia, craniofacial abnormalities, congenital heart defects and Hirschsprung disease. Trisomic cells derived from individuals with Down syndrome possess deficits in SHH signaling, suggesting that overexpression of human chromosome 21 genes may contribute to SHH-associated phenotypes by disrupting normal SHH signaling during development. However, chromosome 21 does not encode any known components of the canonical SHH pathway. Here, we sought to identify chromosome 21 genes that modulate SHH signaling by overexpressing 163 chromosome 21 cDNAs in a series of SHH-responsive mouse cell lines. We confirmed overexpression of trisomic candidate genes using RNA sequencing in the cerebella of Ts65Dn and TcMAC21 mice, model systems for Down syndrome. Our findings indicate that some human chromosome 21 genes, including *DYRK1A*, upregulate SHH signaling, whereas others, such as *HMGN1*, inhibit SHH signaling. Individual overexpression of four genes (*B3GALT5*, *ETS2*, *HMGN1* and *MIS18A*) inhibits the SHH-dependent proliferation of primary granule cell precursors. Our study prioritizes dosage-sensitive chromosome 21 genes for future mechanistic studies. Identification of the genes that modulate SHH signaling may suggest new therapeutic avenues for ameliorating Down syndrome phenotypes.

## Teaser

**Summary:** Human chromosome 21 genes modulate Sonic hedgehog signaling, a pathway essential for organ development that is dysregulated in Down syndrome.

## INTRODUCTION

Down syndrome is a genetically complex condition with trisomy for >200 protein-coding genes contributing to an increased risk of more than 30 phenotypes (Epstein, 2019; Gupta et al., 2016; Moyer and Reeves, 2021). Both trisomy 21 and mutations in the Sonic hedgehog (SHH) signaling pathway predispose affected individuals to cerebellar hypoplasia, holoprosencephaly, microcephaly, autism spectrum disorder, cataracts, cleft palate, Hirschsprung disease, hypotonia, atrial and ventricular septal defects, syndactyly, and polydactyly (Andreu-Cervera et al., 2021; Kallen et al., 1996; Kelley and Hennekam, 2000; Moss et al., 2013; Torfs and Christianson, 1998). This overlap in clinical features prompted the hypothesis that some Down syndrome-associated phenotypes result from aberrant SHH signaling and/or ciliogenesis (Currier et al., 2012).

**Fig. 1.**
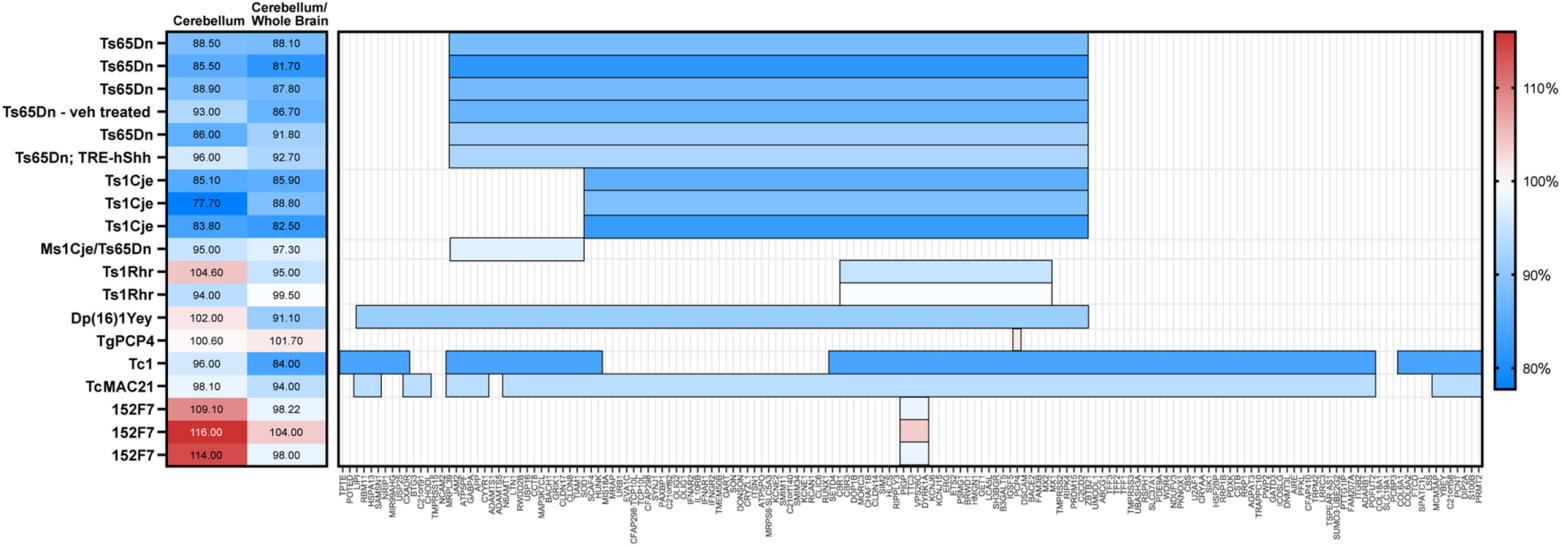
**Comparison of cerebellar phenotypes in Down syndrome mouse models.** (A) Previously published cerebellar volume or cross-sectional area and cerebellar volume or cross-sectional area normalized to that of the whole brain for mouse models are reported as a percentage of the corresponding values in euploid mice. Horizontal bars represent the human chromosome 21 or mouse orthologous regions that are trisomic in each model. Colors reflects the extent of cerebellar hypoplasia, where blue is the most affected and red is the least affected. Several additional studies quantifying cerebellar hypoplasia are generally consistent with these results but do not report these cerebellar measurements (Duchon et al., 2022, 2021; Garcia-Cerro et al., 2018; Ma et al., 2020). Publications referenced in this figure are listed in Table S1.

Cerebellar hypoplasia is one phenotype that is shared between trisomy 21 and ciliopathies such as Joubert syndrome (Joubert et al., 1969). As measured by magnetic resonance imaging, adults with Down syndrome have a disproportionally small cerebellum, even when adjusted for total brain volume (Aylward et al., 1997). During normal development of the cerebellum, SHH acts as the major mitogen for granule cell precursors (Dahmane and Ruiz i Altaba, 1999; Wallace, 1999; Wechsler-Reya and Scott, 1999). Brain samples from adults with Down syndrome have a reduced density of mature cerebellar granule cells, suggesting that trisomic granule cell precursors do not proliferate or differentiate appropriately during development (Baxter et al., 2000). Mirroring the human phenotype, multiple mouse models of Down syndrome, including Tc1, TcMAC21, Dp(16)1Yey, Ts65Dn, and Ts1Cje, also display cerebellar hypoplasia (Fig. 1) (Baxter et al., 2000; Kazuki et al., 2020; Olson et al., 2004; Powell et al., 2016; Starbuck et al., 2014). Although abnormal SHH signaling was first observed in cerebellar cells isolated from the Ts65Dn mouse model of Down syndrome (Roper et al., 2006a), this model is trisomic for ∼60 genes that are not orthologs of human chromosome 21, raising the possibility that non-chromosome 21 orthologs could contribute to the cerebellar phenotypes observed in Ts65Dn mice (Duchon et al., 2022, 2011; Reinholdt et al., 2011).

Studies using cell culture add molecular support to the hypothesis that SHH is dysregulated across trisomic cell types. Compared to control cells, trisomy 21 fibroblasts show reduced expression of the transcription factor GLI1 in response to treatment with the small-molecule SHH agonist SAG, and mouse embryonic fibroblasts (MEFs) isolated from both Dp(16)1Yey and Dp(10)1Yey mouse models possess significantly reduced ciliary localization of the SHH transducer Smoothened (Smo) (Galati et al., 2018; Jewett et al., 2023; McCurdy et al., 2022). A recent study also showed changes in expression of SHH pathway components, including *SHH*, *SMO*, *GLI1*, *GLI2* and *GLI3*, in transitional neural progenitor-like cells derived from human trisomy 21 induced pluripotent stem cells (iPSCs) (Klein et al., 2021). In this study, increasing the concentration of SAG normalized expression of *OLIG2*, a chromosome 21 gene critical for oligodendrocyte development, in human trisomic ‘brain-like’ neural progenitors. Together, these studies demonstrate that dysregulation of the SHH pathway is observed in human trisomy 21 fibroblasts, human trisomy 21 neural progenitors derived from iPSCs, Dp(16)1Yey MEFs and Dp(10)1Yey MEFs.

Although defects in SHH signaling are shared between trisomic models, chromosome 21 does not encode known components of the canonical SHH signaling pathway. Previous attempts to identify chromosome 21 genes involved in SHH signaling have focused on a small subset of candidate genes. The DYRK1A protein kinase has been identified as a modulator of SHH signaling, but returning *Dyrk1a* to disomy was not sufficient to rescue cerebellar volume in Ts65Dn mice (Ehe et al., 2017; Garcia-Cerro et al., 2018; Schneider et al., 2015). Triplication of *APP* has also been proposed to inhibit SHH signaling by upregulating *PTCH1* (Giacomini et al., 2015). Pericentrin (*PCNT*) is a promising candidate gene that, when overexpressed, delays ciliogenesis by altering ciliary trafficking (Galati et al., 2018; Jewett et al., 2023). However, the mouse ortholog of *PCNT* is located on mouse chromosome (MMU) 10, suggesting that additional trisomic genes are responsible for the reduction of ciliary Smo observed in Dp(16)1Yey MEFs and the cerebellar hypoplasia observed in Dp(16)1Yey mice. Additionally, in contrast to human trisomy 21 cerebellar phenotypes, postnatal day (P) 21 Dp(10)1Yey mice do not possess gross changes in cerebellar morphology.

In contrast to these candidate-based approaches, we sought to identify additional modifiers of the SHH pathway using first principles and synthesis of available datasets. We propose that (1) causal genes should be trisomic in mouse models with cerebellar hypoplasia; (2) variation in causal genes may be linked to SHH phenotypes outside of the context of Down syndrome; (3) in the absence of genetic interactions, causal genes should inhibit SHH signaling when overexpressed; and (4) causal genes should be expressed in the relevant cell types and misexpressed in trisomic cells. Here, we integrate data about cerebellar phenotypes collected in mouse models of Down syndrome, Mendelian disorders, a series of *in vitro* cDNA screens and RNA sequencing (RNA-seq) analyses to show that the overexpression of multiple human chromosome 21 genes can modulate SHH signaling. Our findings prioritize four human chromosome 21 genes (*B3GALT5*, *ETS2*, *HMGN1* and *MIS18A*) that are dosage sensitive, expressed in granule cell precursors, and inhibit proliferation when overexpressed in primary granule cell precursors.

## RESULTS

### Comparison of cerebellar phenotypes in Down syndrome mouse models

If a single trisomic gene is sufficient to cause a specific phenotype, individuals with trisomy for that gene will display the phenotype. In humans, this principle has been used to attempt to identify regions associated with intellectual disability, congenital heart anomalies and other – mostly incompletely penetrant – aspects of the syndrome in rare individuals with partial trisomy 21 (Korbel et al., 2009; Korenberg et al., 1994). However, regional brain volume measurements are not available for human subjects with partial trisomy. We instead compared previously reported cerebellar volume or midline cross-sectional area measurements of mouse models at dosage imbalance for different subsets of chromosome 21 genes or the mouse orthologs of these genes (Fig. 1; Table S1). Cerebellar volumes (relative to those of euploid mice) ranged from 78% in Ts1Cje mice to 116% in 152F7 mice.

### Manual annotation of chromosome 21 genes related to SHH and ciliopathies

Disruption of the SHH pathway causes a range of well-characterized phenotypes, including holoprosencephaly, cerebellar hypoplasia, heart defects, skeletal abnormalities, and cancers such as medulloblastoma and basal cell carcinoma. To further understand how overexpression of chromosome 21 genes could affect SHH signaling, we manually annotated chromosome 21 genes associated with hedgehog-related phenotypes through a literature search, the Online Mendelian Inheritance in Man (OMIM) (https://omim.org/) and Mouse Genome Informatics (MGI) (https://www.informatics.jax.org/) databases, and the ciliary/centrosome database Cildb v3.0 (http://cildb.i2bc.paris-saclay.fr/) (Table S2). Of the 44 chromosome 21 genes with associated phenotypes in OMIM, four genes (*CFAP298*, *CFAP410*, *PCNT* and *RSPH1*) encode proteins involved in ciliogenesis. Mutations in an additional 12 genes (*CSTB*, *DSCAM*, *JAM2*, *KCNJ6*, *OLIG1*, *OLIG2*, *PRDM15*, *PSMG1*, *SOD1*, *SON*, *TRAPPC10* and *WDR4*) are associated with cerebellar phenotypes or holoprosencephaly in humans or in mouse models.

### Primary screen for chromosome 21 cDNAs that affect SHH signaling

**Fig. 2.**
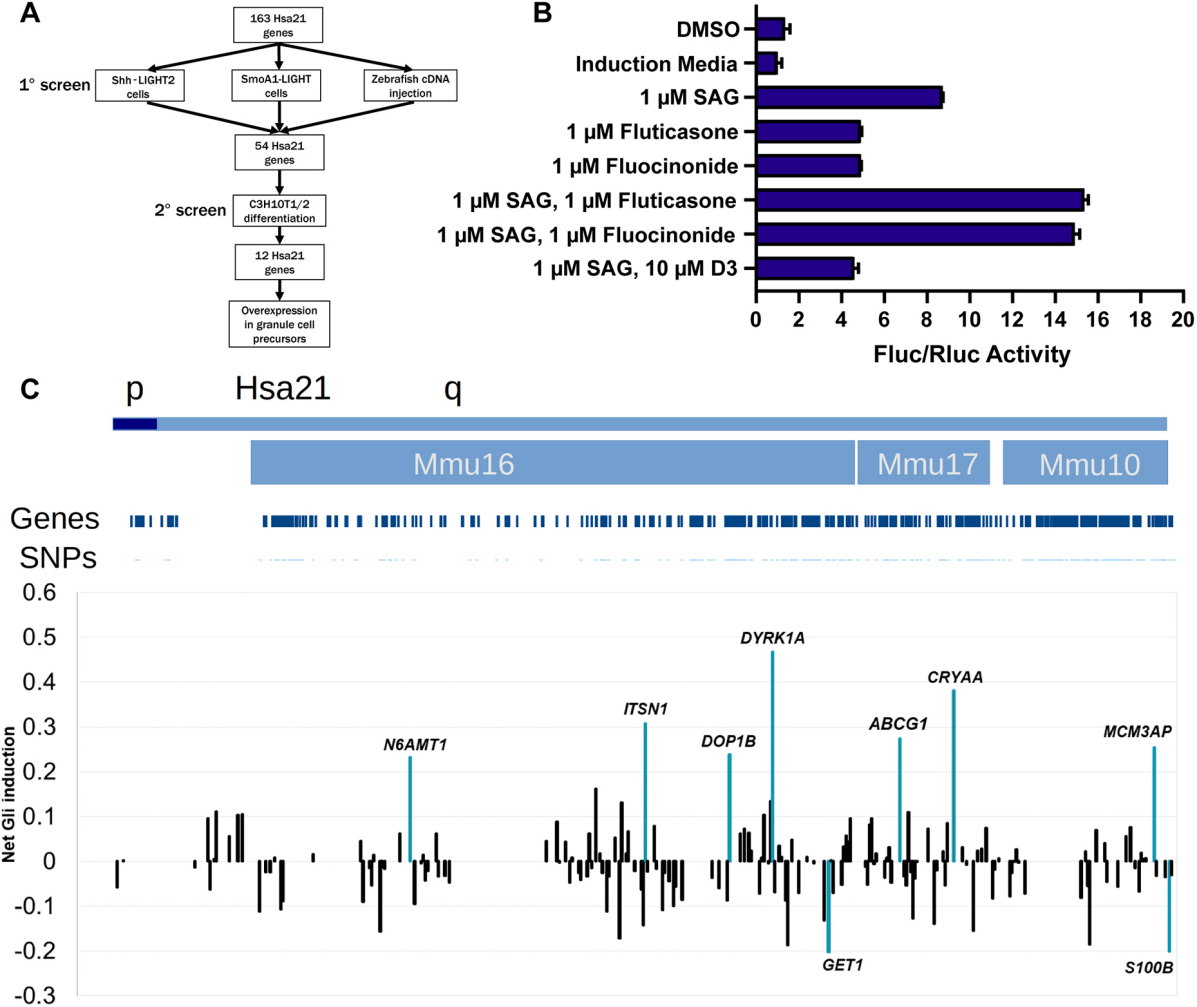
**Overexpression of human chromosome 21 cDNAs in Shh-LIGHT2 cells.** (A) Screening strategy for chromosome 21 cDNAs in Shh-LIGHT2 and SmoA1-LIGHT cell lines, zebrafish embryos, the C3H10T1/2 mesenchymal stem cell line, and primary granule cell precursors. (B) Fluc/Rluc activity in Shh-LIGHT2 cells exposed to SAG, the glucocorticoids fluocinonide and fluticasone, and vitamin D3, normalized to that of the induction media control (*n*=2 independent experiments with 12 technical replicates per treatment). All graphs show mean±s.d. unless otherwise noted. (C) Shh-LIGHT2 cells transfected with expression constructs for 163 chromosome 21 cDNAs and treated with SAG to induce SHH signaling (≥8 technical replicates per cDNA; see Table S4 for wells per cDNA). Averaged Fluc/Rluc activity for each gene across the Shh-LIGHT2 screen was scaled to 0 to show signal deflections from baseline. Values less than zero represent loci that decrease SAG-induced activation of the SHH signaling pathway. The net activity of the 8×GliBS reporter for each cDNA is plotted in chromosomal order according to the sequence along the proximal-distal length of human chromosome 21. Orthologous regions on mouse chromosomes 16, 17, and 10 are provided for additional context. The labeled cDNAs increased or decreased Fluc/Rluc activity by more than two standard deviations.

Although several chromosome 21 genes have previously been associated with SHH signaling, most annotations derive from loss-of-function mutations rather than overexpression. To identify genes of which overexpression is sufficient to modulate SHH signaling, we designed a multilevel screen in zebrafish (Edie et al., 2018) and in four SHH-responsive cell types (Fig. 2A). We first screened a library of 163 human chromosome 21 cDNAs selected for high homology to mouse genes (Table S3) in two well-established SHH-responsive reporter cell lines: mouse Shh-LIGHT2 cells, which express firefly luciferase (Fluc) from the SHH-responsive promoter of *Gli1* (8×GliBS-FL; Sasaki et al., 1997) and Renilla luciferase (Rluc) from a constitutive promoter (pRL-TK, Promega), and mouse SmoA1-LIGHT cells, which are based on Shh-LIGHT2 cells but also possess an oncogenic mutation in Smo (W539L) that activates SHH signaling in the absence of pharmacological stimulation (Taipale et al., 2000).

**Fig. 3.**
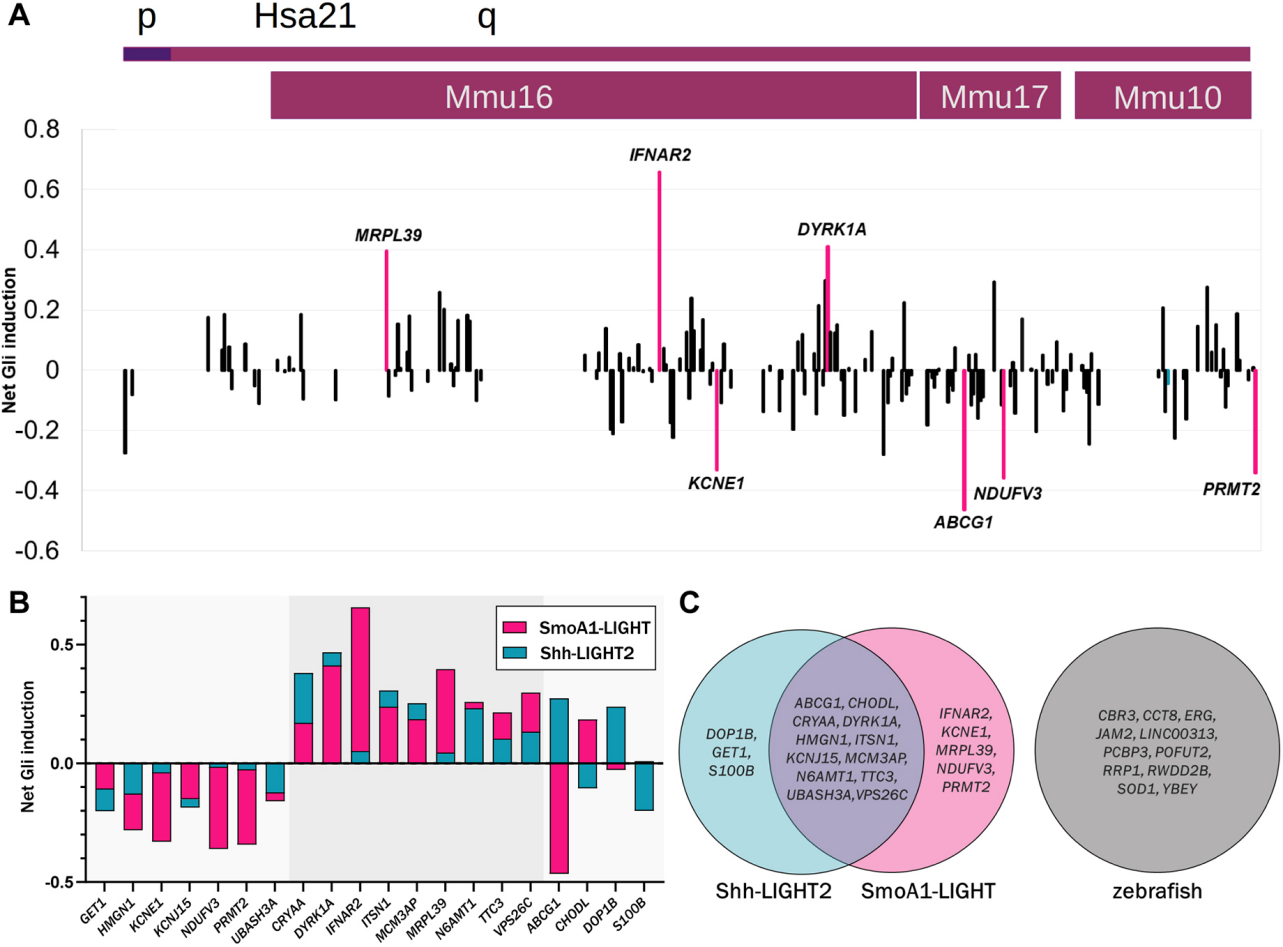
**Overexpression of human chromosome 21 cDNAs in SmoA1-LIGHT cells.** (A) SmoA1-LIGHT cells transfected with expression constructs for 163 human chromosome 21 cDNAs (≥8 technical replicates per cDNA; see Table S5 for wells per cDNA). Averaged Fluc/Rluc activity for each gene across the SmoA1-LIGHT screen was scaled to zero to show signal deflections from baseline. The labeled cDNAs increased or decreased Fluc/Rluc activity by more than two standard deviations. (B) Comparison of net reporter induction after overexpression of twenty cDNAs identified in SmoA-LIGHT and Shh-LIGHT2 screens. Sixteen cDNAs have the same direction of effect in both screens, whereas four cDNAs have opposite effects. The gray highlight indicates cDNAs that increase Fluc/Rluc activity in both cell lines. (C) Comparison of cDNAs identified in two luciferase assays and a previous screen in developing zebrafish embryos (Edie et al., 2018).

Shh-LIGHT2 cells responded robustly to the hedgehog agonists fluocinonide, fluticasone and SAG, whereas vitamin D3 inhibited SAG-induced reporter activity (Fig. 2B). Transient overexpression of nine human genes increased or decreased the ratio of Fluc activity to Rluc activity by more than two standard deviations (z≤−2 or z≥2) in Shh-LIGHT2 cells treated with SAG (Table S4). Overexpression of *ABCG1*, *CRYAA*, *DOP1B*, *DYRK1A*, *ITSN1*, *MCM3AP* and *N6AMT1* activated SHH signaling, whereas overexpression of *GET1* and *S100B* inhibited signaling (Fig. 2C). In SmoA1-LIGHT cells, overexpression of *DYRK1A*, *IFNAR2* and *MRPL39* increased SHH signaling by more than two standard deviations, and overexpression of *ABCG1*, *KCNE1*, *NDUFV3* and *PRMT2* inhibited SHH signaling (Fig. 3A; Table S5). We also identified an additional six human genes that modulated SHH signaling by more than one standard deviation in both screens: *CHODL*, *HMGN1*, *KCNJ15*, *TTC3*, *UBASH3A* and *VPS26C*. Of the twenty total human genes identified in Shh-LIGHT2 or SmoA1-LIGHT screens, sixteen affected SHH signaling in the same manner in both cell lines: overexpression of *GET1*, *HMGN1*, *KCNE1*, *KCNJ15*, *NDUFV3*, *PRMT2* and *UBASH3A* inhibited SHH signaling; overexpression of *CRYAA*, *DYRK1A*, *IFNAR2*, *ITSN1*, *MCM3AP*, *MRPL39*, *N6AMT1*, *TTC3* and *VSP26C* upregulated SHH signaling; and overexpression of *ABCG1*, *CHODL*, *DOP1B* and *S100B* showed discordant effects in the two cell lines (Fig. 3B). We previously screened this human chromosome 21 cDNA library in developing zebrafish and identified eleven genes that caused gross morphological defects or lethality when overexpressed; seven of these genes affected development of structures that are substantially influenced by or dependent on SHH signaling (Edie et al., 2018). However, there was no overlap between any of these eleven genes and the twenty genes prioritized by the luciferase assays (Fig. 3C).

We compared the results of our cDNA overexpression screens to four previously reported genome-wide siRNA knockdown and CRISPR knockout screens in NIH3T3-derived cell lines containing the 8×GliBS reporter (Fig. S1; Table S6). Neither Shh-LIGHT2 nor SmoA1-LIGHT screens showed a significant correlation with two siRNA screens performed in NIH3T3-Shh-FL cells, which produce SHH endogenously (Jacob et al., 2011). However, our Shh-LIGHT2 screen showed a weak negative correlation with two CRISPR knockout screens in NIH3T3 cells treated with the N-terminal domain of Shh, suggesting that knockout and overexpression of some chromosome 21 genes may have opposing effects on SHH signaling (Breslow et al., 2018; Pusapati et al., 2018b). Of the 31 candidate genes identified by our cDNA and zebrafish screens, *DYRK1A*, *GET1*, *MCM3AP*, *PCBP3* and *POFUT2* were identified in one or more of the four knockdown/knockout screens.

### Secondary screen using a functional cell-based assay of osteoblast differentiation

**Fig. 4.**
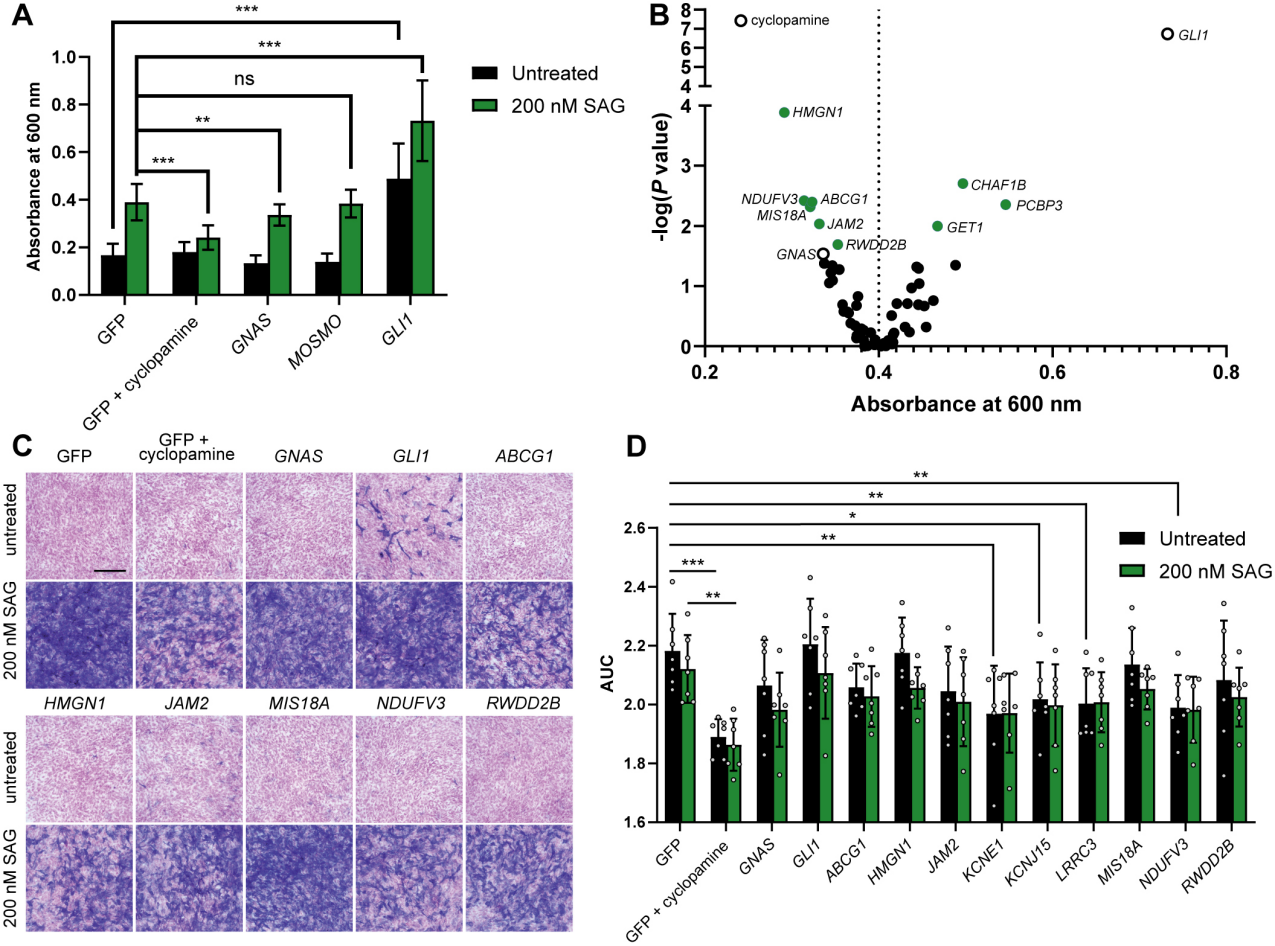
**Overexpression of human chromosome 21 genes affects osteoblast differentiation of C3H10T1/2 cells.** (A) Overexpression of *GLI1* promotes osteoblast differentiation in the presence or absence of SAG, whereas treatment with cyclopamine or overexpression of *GNAS* inhibits SAG-induced osteoblast differentiation (*n*=20) (two-tailed unpaired Student's *t*-test). (B) Quantification of alkaline phosphatase activity in C3H10T1/2 cells transfected with human chromosome 21 cDNAs and treated with SAG (*n*=20). Multiple comparisons were corrected for by controlling the false discovery rate; green circles denote cDNAs with q<0.1; open circles denote controls (Kruskal–Wallis test followed by Dunn's post hoc test). (C) Representative images of alkaline phosphatase staining in C3H10T1/2 cells transfected with human chromosome 21 cDNAs and counterstained with Nuclear Fast Red (*n*=3). Scale bar: 100 μm. (D) MTT viability assay in C3H10T1/2 cells transfected with chromosome 21 cDNAs (*n*=7). The *y*-axis represents area under the curve (AUC) values of cell viability 48, 72 and 96 h (*n*=7 for each) after transfection (two-way ANOVA followed by Fisher's least significant difference test). Differences reported as statistically significant have q<0.05. ns, not significant; *P<0.05; **P<0.01; ***P<0.001.

Based on our primary screen, we selected 54 human chromosome 21 genes for further characterization in a functional cell-based assay (Table S3). The mouse C3H10T1/2 mesenchymal stem cell line undergoes SHH-dependent differentiation into osteoblasts and has been used to identify agonists and antagonists of the SHH signaling pathway (Nakamura et al., 1997, 2015; Roudaut et al., 2011). We transfected C3H10T1/2 cells with candidate human cDNAs and quantified alkaline phosphatase activity, an early marker of osteoblast differentiation. In the absence of SAG treatment, overexpression of *GLI1* was sufficient to induce osteoblast differentiation (Fig. 4A). Stimulation of osteoblast differentiation by 200 nM SAG was inhibited by co-treatment with 2 μM cyclopamine and by overexpression of the heterotrimeric G-protein subunit GαS (*GNAS*), which inhibits SHH signaling via protein kinase A (PKA) (Pusapati et al., 2018a,b). Overexpression of the previously identified regulator of SHH signaling *MOSMO* had no effect on alkaline phosphatase activity, whereas overexpression of *GLI1* further induced osteoblast differentiation even in the presence of SAG (Pusapati et al., 2018b).

In C3H10T1/2 cells treated with SAG, overexpression of six human chromosome 21 cDNAs (*ABCG1*, *HMGN1*, *JAM2*, *MIS18A*, *NDUFV3* and *RWDD2B*) significantly reduced osteoblast differentiation compared to that of control cells, indicating that overexpression of these cDNAs attenuated SHH signaling (Fig. 4B; Fig. S2). Overexpression of three human chromosome 21 cDNAs (*CHAF1B*, *GET1* and *PCBP3*) significantly increased osteoblast differentiation compared to that of control cells. Staining of a subset of cells overexpressing human cDNAs for alkaline phosphatase activity confirmed inhibition of osteoblast differentiation and suggested a possible reduction in cell density following transfection of some cDNAs (Fig. 4C). Because reduced viability could affect osteoblast differentiation independently of SHH signaling, we assessed cell viability at three time points post transfection using a 3-(4, 5-dimethylthiazolyl-2)-2, 5-diphenyltetrazolium bromide (MTT) assay. Both cDNA [f(12, 156)=5.327, *P*<0.0001] and SAG treatment [f(1, 156)=6.474, *P*=0.0119] had a significant effect on viability, but the interaction between these terms was not significant (Fig. 4D; Fig. S2). In untreated cells, overexpression of *KCNE1*, *KCNJ15*, *LRRC3* and *NDUFV3* and treatment with cyclopamine reduced cell viability compared to that of control cells. In cells treated with SAG, only cyclopamine treatment significantly affected viability.

### Expression of candidate genes in the developing cerebellum

**Fig. 5.**
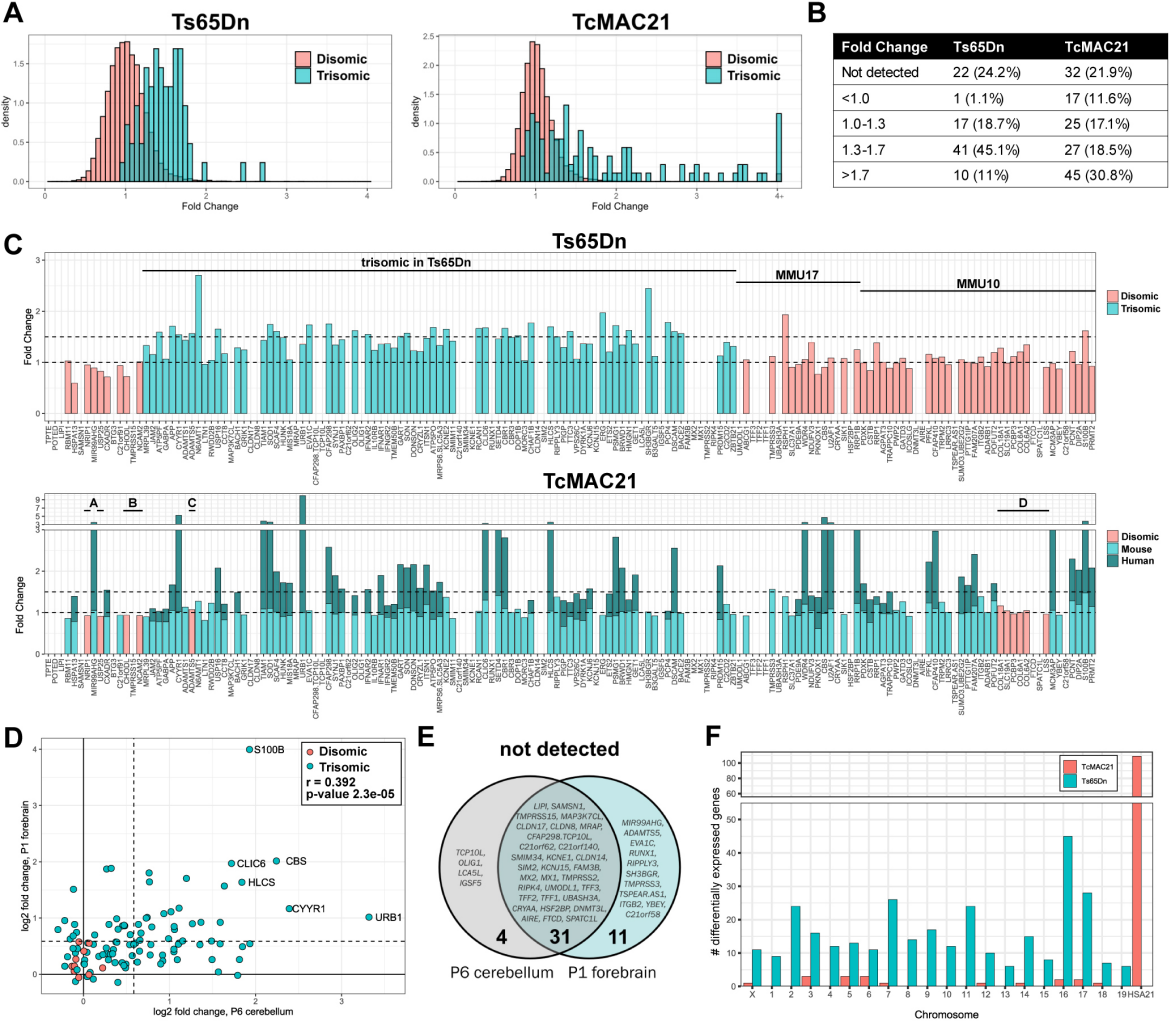
**Expression pattern of chromosome 21 genes and their mouse orthologs in Ts65Dn and TcMAC21 cerebellum.** (A) Density histograms of disomic (salmon) and trisomic (teal) fold changes in Ts65Dn and TcMAC21 cerebella (*n*=4 Ts65Dn, 4 Ts65Dn euploid littermates, 4 TcMAC21, 4 TcMAC21 euploid littermates). The plots represent 13,807 detectable transcripts. (B) Trisomic gene fold changes binned by expression levels. (C) Fold changes of human chromosome 21 genes and their mouse orthologs arranged in chromosomal order from proximal to distal. Human chromosome 21 orthologs are located on mouse chromosome 16 (MMU16), MMU17 and MMU10. For TcMAC21, teal represents the proportion of length-normalized reads contributed by mouse copies and dark teal represents reads derived from the human chromosome. Four previously reported deletions are labeled ‘A’ through ‘D’. Five human genes that were detected in TcMAC21 but have no expression of mouse orthologs for normalization (*POTED*, *BTG3*, *RUNX1*, *C21orf58* and *TSPEAR-AS1*) are excluded. (D) Scatterplot of log_2_(fold change) values for human chromosome 21 gene expression in TcMAC21 P6 cerebellum and P1 forebrain (Kazuki et al., 2020). Pearson correlation coefficient *R*=0.392 and *P*=2.3×10^−5^. (E) Human chromosome 21 transcripts not detected in the P6 cerebellum, P1 forebrain or both. (F) Chromosomal locations of differentially expressed genes in Ts65Dn and TcMAC21 cerebellum. Trisomic genes are located on MMU16 and MMU17 in Ts65Dn mice and Hsa21 in TcMAC21 mice.

To determine whether candidate genes are expressed in a SHH-responsive tissue relevant to Down syndrome-associated cerebellar hypoplasia, we performed RNA-seq on P6 cerebella collected from Ts65Dn (*n*=4 trisomic and 4 euploid littermates) and TcMAC21 (*n*=4 trisomic and 4 euploid littermates) pups. TcMAC21 mice carry a nearly intact copy of the long arm of human chromosome 21, including 93% of the protein-coding human genes, as the HSA21q-MAC mouse artificial chromosome. At this stage of development, the cerebellum is composed predominantly of proliferating granule cell precursors and differentiating granule cells (Rosenberg et al., 2018; van Essen et al., 2020). We previously found that granule cell precursors isolated from P6 Ts65Dn pups respond less to the mitogenic effects of SHH than euploid cells, and by P6, the cerebellar cross-sectional area is significantly reduced in Ts65Dn pups (Roper et al., 2006a). For TcMAC21 samples, length-normalized counts for human chromosome 21 transcripts were added to counts for corresponding mouse orthologs and compared to euploid counts. Trisomic genes were overexpressed by an average of 1.45±0.29 (indicated as mean±s.d.) in Ts65Dn mice and 1.81±1.18 in TcMAC21 mice compared to euploid (Fig. 5A). The majority of trisomic genes with detectable expression in Ts65Dn mice had fold changes between 1.3 and 1.7, whereas TcMAC21 samples had a higher proportion of trisomic genes with fold changes above 1.7 (Fig. 5B). Arranged by chromosomal position, expression patterns were consistent with the previously reported breakpoint of the Ts65Dn 17^16^ chromosome and the four deletions reported in the TcMAC21 HSA21q-MAC hybrid chromosome (Fig. 5C) (Duchon et al., 2011; Kazuki et al., 2020). Expression of human chromosome 21 genes in the TcMAC21 cerebellum was positively correlated with previously published P1 forebrain expression levels (r=0.39, *P*=2.3×10^−5^) (Fig. 5D), and 31 human genes were not detected in the TcMAC21 P1 forebrain or P6 cerebellum (Fig. 5E).

We also identified differential expression of disomic genes in both Ts65Dn and TcMAC21 models (Fig. 5F; Fig. S3; Tables S7 and S8). Although expression levels in Ts65Dn and TcMAC21 cerebella were positively correlated (r=0.529 and *P*=2.2×10^−16^), only two disomic genes, *Lrch4* and *Snhg11*, were significantly differentially expressed in both models using a false discovery rate of 0.05 (Fig. S3). Gene Ontology and gene set enrichment analyses of differentially expressed genes in Ts65Dn samples suggested changes in gene expression related to nervous system development, higher mental function and cholesterol biosynthesis (Figs S4 and S5; Table S9). Ts65Dn samples also showed reduced expression of mitotic and cell cycle pathway-related genes and increased expression of genes related to protein translation initiation and elongation (Fig. S5). The trisomic chromatin modifiers and remodelers *Chaf1b*, *Hmgn1*, *Setd4* and *Brwd1* were significantly upregulated, and the non-trisomic epigenetic regulators *Rps6ka5*, *Rere*, *Brd4*, *Kdm7a* and *Top2a* were dysregulated (Fig. S6). Genes encoding elements of the Polycomb repressive complex (*Mbd6*, *Pcgf2* and *Auts2*) and the SWI/SNF complex (*Arid1a*, *Arid1b* and *Bicra*) were also upregulated.

### Integration of expression and SHH screen data to prioritize candidate genes

**Fig. 6.**
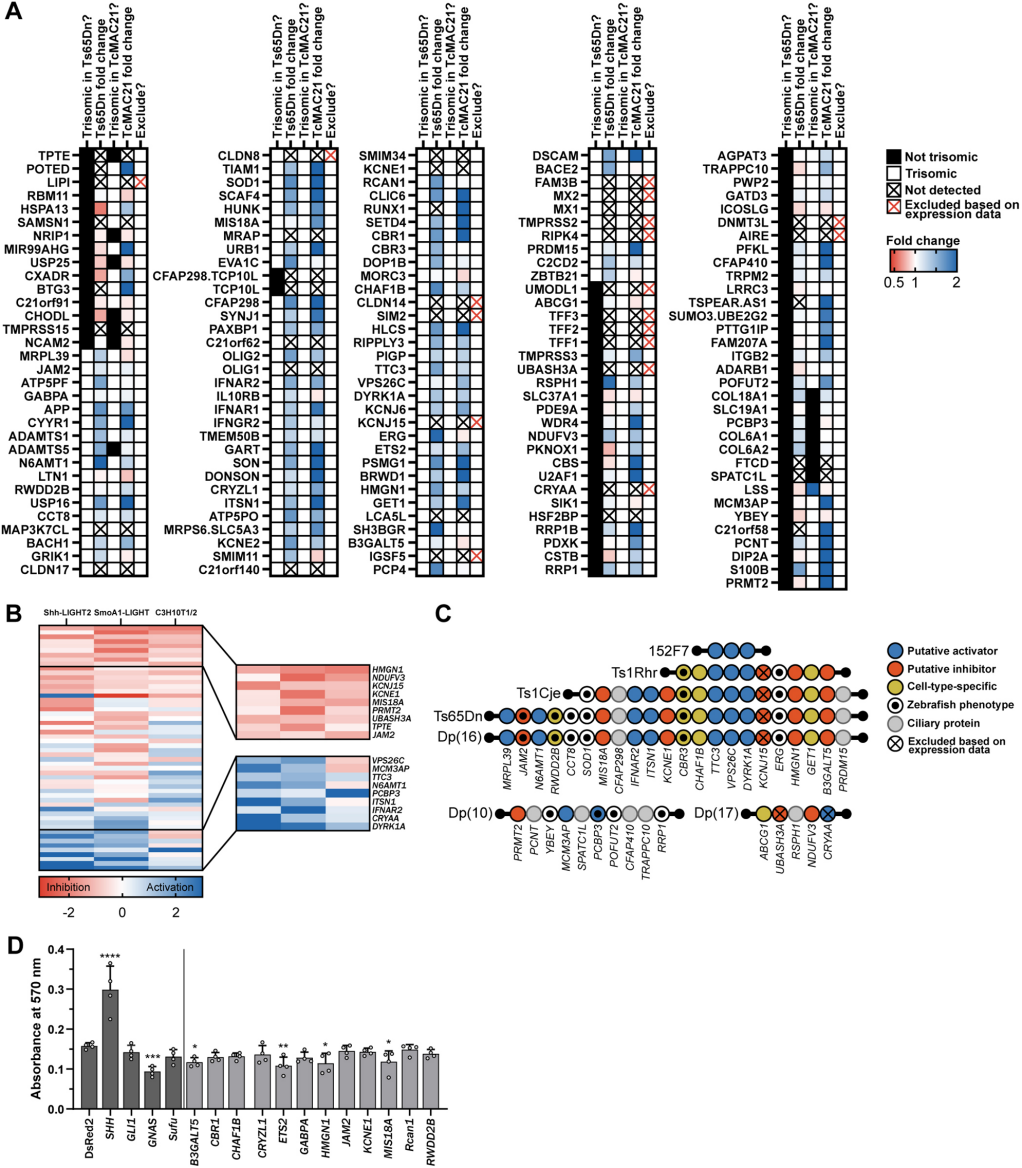
**Prioritization of candidate cDNAs and overexpression in primary granule cell precursors.** (A) Summary of expression data in the developing cerebellum. Black boxes indicate genes that are not trisomic in Ts65Dn and TcMAC21 mouse models, and white boxes indicate genes that are trisomic in these models. Fold change in gene expression is indicated by color, with red signifying decreased expression and blue signifying increased expression. Transcripts with black crosses were not detected in our RNA-seq dataset, and transcripts with red crosses were excluded based on our expression data, expression in the BrainSpan Atlas of the Developing Human Brain and single cell RNA-seq data from euploid mouse granule cell precursors and granule cells. (B) Comparison of the effects of 54 human chromosome 21 cDNAs in Shh-LIGHT2, SmoA1-LIGHT and C3H10T1/2 screens. cDNAs are sorted by average z-score, with red signifying inhibition and blue signifying activation of the SHH pathway. The inset shows top- and bottom-ranked cDNAs. (C) Chromosomal locations of the mouse orthologs of candidate cDNAs in Down syndrome mouse models. *LINC00313* (*C21ORF84*) and *TPTE* are not shown. *LINC00313*, which was identified in the zebrafish screen, is a human-specific gene and not present in the listed mouse models. *TPTE* is located on the short arm of human chromosome 21 and has a putative homolog on mouse chromosome 8. (D) Lentiviral overexpression of candidate genes inhibits proliferation of granule cell precursors treated with 6 nM SAG and pulsed with EdU for 24 h (*n*=4). Dark gray bars indicate control cDNAs and light gray bars indicate human chromosome 21 cDNAs. *P<0.05; **P<0.01; ***P<0.001; ****P<0.0001 (one-way ANOVA followed by Fisher's least significant difference test). Differences reported as statistically significant have q<0.05.

We next integrated expression data with our primary and secondary SHH screen data. Leading candidate genes should be expressed in the developing cerebellum, be trisomic in mouse models with cerebellar hypoplasia, and consistently inhibit SHH across *in vitro* screens. Eighteen genes were not detected in our RNA-seq data, had fragments per kilobase of exon per million mapped (FPKM) values <1 in 13 human cerebellar samples acquired from 12 weeks to 4 months post conception (BrainSpan Atlas of the Developing Human Brain; https://www.brainspan.org/), and had transcripts per million (TPM) values <1 in mouse P2 and P11 granule cell precursor and granule cell populations (Fig. 6A; Table S10) (Miller et al., 2014; Rosenberg et al., 2018). Although these 18 genes may contribute to dysregulated SHH signaling in other tissues, such as the heart or craniofacial skeleton, they appear as unlikely candidates for cerebellar hypoplasia.

Synthesis of data from our primary luciferase screens and secondary C3H10T1/2 screen also revealed candidate genes with the most consistent effects across cell lines (Fig. 6B). For example, overexpression of *HMGN1* consistently inhibited SHH, whereas overexpression of *DYRK1A* consistently activated SHH. Overexpression of most cDNAs showed relatively consistent effects across cell types, but overexpression of some cDNAs, such as *ABCG1* and *GET1*, showed strong but discordant effects across screens. We mapped candidate cDNAs to mouse models and identified a subset of six genes that appeared to inhibit SHH across screens and have mouse orthologs located on mouse chromosome 16 (Fig. 6C). Putative activators of SHH with mouse orthologs on chromosome 16 (*DYRK1A*, *IFNAR2*, *ITSN1*, *MRPL39*, *N6AMT1*, *TTC3* and *VPS26C*) may provide compensatory effects, whereas putative inhibitors of SHH with mouse orthologs on chromosomes 10 and 17 (*NDUFV3*, *PRMT2* and *UBASH3A*) may inhibit SHH via a mechanism independent of dysregulated SHH signaling in Ts65Dn and Dp(16)1Yey cells.

### Overexpression of four candidate genes inhibits proliferation of primary granule cell precursors

To evaluate top candidate cDNAs in a context relevant to cerebellar hypoplasia, we cloned 12 human chromosome 21 cDNAs into lentiviral vectors, overexpressed them in primary euploid granule cell precursors, and quantified proliferation via incorporation of 5-ethynyl-2′-deoxyuridine (EdU). Candidate cDNAs were selected based on literature evidence, performance in cellular and zebrafish screens, chromosomal position, and expression level (Table S3). As expected, overexpression of *SHH* itself significantly increased proliferation, and overexpression of *GNAS*, which has been identified as a tumor suppressor gene in the SHH subtype of medulloblastoma, significantly inhibited proliferation (Fig. 6D) (He et al., 2014). Of the 12 human chromosome 21 cDNAs, overexpression of four (*B3GALT5*, *ETS2*, *HMGN1* and *MIS18A*) significantly reduced proliferation compared to overexpression of DsRed2. These results suggest that at least some of the candidate cDNAs identified in the luciferase, zebrafish and C3H10T1/2 screens also modulate SHH signaling in the developing cells of the cerebellum.

## DISCUSSION

Our data provide novel insights into the complex genetic architecture of aberrant SHH signaling in Down syndrome. We previously showed that a reduced mitogenic response to SHH underlies cerebellar hypoplasia in Ts65Dn mice but lacked a clear understanding of which trisomic genes contribute to this phenotype (Roper et al., 2006a). In this study, we prioritized human chromosome 21 genes that consistently modulate SHH signaling in a variety of cellular contexts and identified four genes (*B3GALT5*, *ETS2*, *HMGN1* and *MIS18A*) that impair the proliferation of cerebellar granule cell precursors when overexpressed.

In contrast to previous hypothesis-driven approaches, our study provides quantitative data about the individual effects of nearly all human chromosome 21 protein-coding genes conserved between human and mouse. Although trisomy of any chromosome has the potential to impair proliferation via aneuploidy stress (Zhu et al., 2018), our data show that overexpression of specific human chromosome 21 genes inhibits the proliferation of granule cell precursors. In fact, overexpression of 127 of the 163 human cDNAs had no effect in the luciferase, zebrafish, C3H10T1/2 or granule cell precursor assays, indicating that cDNA overexpression does not have a non-specific effect on SHH signaling and, barring genetic interactions, excludes these genes as candidates. Lack of effect in our SHH screens does not eliminate them as contributors to a general destabilization of the trisomic transcriptome, nor does it consider effects in the context of a transcriptome destabilized by trisomy for individually benign trisomic genes.

### Severity of cerebellar hypoplasia in mouse models depends on trisomic gene content

Our results provide evidence for why Down syndrome mouse models present with variable severities of cerebellar hypoplasia (Fig. 1). Ts65Dn mice and Ts1Cje mice possess a similar reduction of cerebellar volume normalized to total brain volume (Aldridge et al., 2007; Baxter et al., 2000; Das et al., 2013; Gao et al., 2021; Laffaire et al., 2009; Olson et al., 2004; Wu et al., 2021a). Ms1Cje/Ts65Dn mice, which are not trisomic for 80 of the genes overrepresented in Ts65Dn mice, do not show substantial cerebellar hypoplasia, although only three such trisomic animals have been analyzed (Olson et al., 2004). Ts1Rhr mice show more subtle cerebellar hypoplasia than Ts65Dn and Ts1Cje mice (Aldridge et al., 2007; Olson et al., 2007). Comparing these four models suggests that at least one gene in the region that is trisomic in both Ts1Cje and Ts65Dn mice but is not trisomic in Ts1Rhr mice (*Sod1* to *Setd4* and *Ripk4* to *Zbtb21*) contributes to cerebellar hypoplasia. An additional gene or genes may contribute to the mild cerebellar hypoplasia observed in Ts1Rhr mice (*Cbr1* to *Mx2*). 152F7 mice, which contain a yeast artificial chromosome with human *PIGP*, *TTC3*, *VPS26C* and *DYRK1A*, show increased cerebellar volume relative to that of control mice, suggesting that overexpression of this region provides a compensatory effect (Sebrie et al., 2008). Although Tc1 mice also display cerebellar hypoplasia, interpreting the genetic contributions to this phenotype is challenging due to mosaicism and complex rearrangements in the Tc1 human chromosome (Gribble et al., 2013; Powell et al., 2016). Overexpression of *PCP4*, encoding Purkinje cell protein 4, does not affect cerebellar volume (Mouton-Liger et al., 2014).

The results from our SHH screen are consistent with a model in which *B3galt5*, *Ets2*, *Hmgn1* and *Mis18a* contribute to the severe cerebellar hypoplasia observed in Ts65Dn and Ts1Cje mice, *Hmgn1* and *Ets2* contribute to the milder hypoplasia in Ts1Rhr mice, and *Ttc3*, *Vps26c* and *Dyrk1a* provide a compensatory effect in Ts65Dn, Ts1Cje, Ts1Rhr and 152F7 mice. Compensatory effects may also explain why relatively mild cerebellar hypoplasia was reported in Dp(16)1Yey and in TcMAC21 mice, despite these models containing more trisomic genes than either Ts65Dn or Ts1Cje (Kazuki et al., 2020; Starbuck et al., 2014). For example, overexpression of *MCM3AP*, a putative activator of SHH signaling and MMU10 ortholog, could provide a compensatory effect in the TcMAC21 cerebellum.

Interpreting the contributions of trisomic genes to cerebellar hypoplasia is further complicated by differences in expression of trisomic genes between models. TcMAC21 and Tc1 models rely on appropriate function of human DNA regulatory elements in mouse cells, and gene expression may differ between models with segmental duplications [e.g. Dp(16)1Yey and Ts1Rhr] versus freely segregating chromosomes (Ts65Dn) (Aziz et al., 2018). For example, we found that *B3GALT5*, a putative inhibitor of SHH signaling, has a fold change of 0.92 in TcMAC21, despite this model having two copies of mouse *B3galt5* and one copy of human *B3GALT5.* Moreover, although our SHH screen and RNA-seq data support an oligogenic or polygenic explanation for cerebellar hypoplasia in mouse models, testing this hypothesis by returning candidate genes to disomy would be technically challenging owing to difficult husbandry, relatively subtle phenotypes and high interindividual variability (Roper and Reeves, 2006; Roper et al., 2006b; Shaw et al., 2020).

### Limitations of the Ts65Dn mouse model

Mapping of the Ts65Dn translocation breakpoint revealed that Ts65Dn mice are trisomic for ∼40 protein-coding genes that are not orthologs of human chromosome 21 genes (Duchon et al., 2011; Reinholdt et al., 2011). These ‘extra’ trisomic genes may contribute to phenotypes observed in Ts65Dn mice, and our RNA-seq data identified significant overexpression of ∼15 trisomic mouse chromosome 17 genes (Table S7; Fig. S3A). Several of these genes, including *Arid1b*, *Gtf2h5*, *Ezr*, *Rsph3b*, *Rsph3a* and *Pde10a*, have known or predicted roles in neurodevelopment and may influence Ts65Dn-specific brain and behavioral phenotypes (Fig. S6).

The Herault group recently removed the trisomic mouse chromosome 17 genes from Ts65Dn mice using CRISPR/Cas9 engineering (Duchon et al., 2022; Guedj et al., 2023). The resulting Ts66Yah line will help to resolve the contributions of trisomic genes to individual phenotypes, although phenotypes are generally attenuated in animals with fewer trisomic genes. Furthermore, the strains ‘1924’ and ‘5252’ of Ts65Dn show differences in cerebellar cellularity, and strain differences between the 1924 Ts65Dn and the 5252-derived Ts66Yah may confound direct comparison of cerebellar phenotypes between these lines (Shaw et al., 2020). Nonetheless, abnormal SHH signaling in human trisomy 21 cells indicates that chromosome 21 genes and their orthologs are sufficient to modulate the SHH pathway.

### Gene dosage effects on SHH signaling

The molecular mechanisms by which chromosome 21 genes inhibit SHH signaling merit additional exploration. It is surprising that overexpression of several known ciliary genes (*CFAP298*, *CFAP410*, *RSPH1*, *SPATC1L* and *TRAPPC10*) had no effect in our luciferase screens, consistent with a previous report that overexpression of *CFAP298*, *CFAP410* and *TRAPPC10* did not alter cilia formation (Galati et al., 2018). Instead, we identified a number of regulators of mitosis and chromatin structure, including *CHAF1B*, *HMGN1*, *MCM3AP*, *MIS18A* and *N6AMT1*; two involved in endocytosis, *ITSN1* and *VPS26C*; and a cholesterol transporter, *ABCG1*. These results suggest that rather than inhibiting the canonical SHH pathway directly, overexpression of some human chromosome 21 genes may affect cell state, epigenetic regulation and progression through the cell cycle. This hypothesis is supported by differential expression of chromatin regulators in Ts65Dn cerebellum and gene set enrichment analysis showing reduced expression of transcripts encoding mitotic proteins.

A promising candidate for disruption of normal chromatin structure is *HMGN1*, which inhibited SHH signaling when overexpressed in Shh-LIGHT2 and SmoA1-LIGHT cells, C3H10T1/2 cells, and primary granule cell precursors. In our previous zebrafish screen, only nine of 120 embryos survived injection of 50 pg *HMGN1* mRNA, and of the nine surviving embryos, four had missing melanocytes (Edie et al., 2018). However, this finding was not reproduced in a secondary screen. In *Xenopus laevis* embryos, injection of HMGN1 protein causes body axis curvature, cyclopia and microcephaly, which are all phenotypes associated with aberrant SHH signaling (Korner et al., 2003). *hmgn1* is expressed in the pharyngeal arches of *Xenopus* embryos, and knockdown of *hmgn1* disrupts cranial neural crest streams, resulting in hypoplastic craniofacial cartilage (Ihewulezi and Saint-Jeannet, 2021).

*HMGN1* encodes a non-histone chromosomal protein that competes for binding with histone H1 (Postnikov and Bustin, 2010). Binding of HMGN1 reduces chromatin compaction and is associated with lineage-specific regulatory elements (He et al., 2018). HMGN1 expression levels are correlated with the transition from proliferation to differentiation in stem cells, and in primary rat osteoblasts, *Hmgn1* is preferentially expressed in proliferating cells, with a decline in expression at the onset of mineralization (Shakoori et al., 1993). Loss of *Hmgn1* and *Hmgn2* in MEFs increases the efficiency of reprogramming into iPSCs, suggesting that HMGN proteins help to stabilize cell identity (He et al., 2018). In B cells, *HMGN1* overexpression results in a loss of H3K27me3, a gain of H3K27ac and a global increase in transcription (Lane et al., 2014; Mowery et al., 2018). HMGNs act upstream of *Olig1* and *Olig2* during oligodendrocyte differentiation, indicating a possible interplay between SHH signaling, *HMGN1* and *OLIG1/OLIG2* during neurodevelopment (Deng et al., 2017; Klein et al., 2021). Together, the known roles of HMGN1 suggest that HMGN1 overexpression could disrupt the proliferation of granule cell precursors by altering epigenetic marks, disrupting the balance of proliferation and differentiation (e.g. precocious differentiation), or promoting differentiation along an alternative cell state trajectory, such as differentiation into astrocytes (Okano-Uchida et al., 2004).

The *B3GALT5* gene encodes the N-acetylglucosamine-β-1,3-galactosyltransferase protein, and overexpression of *B3GALT5* inhibited the proliferation of granule cell precursors in our screen. The function of the B3GALT5 protein is most well-studied in colon, pancreatic and breast cancers, in which B3GALT5 catalyzes the synthesis of the sialyl Lewis-a antigen (Engle et al., 2019; Isshiki et al., 1999; Liao et al., 2021). B3GALT5 is also involved in the transition of human embryonic stem cells (hESCs) from a primed to a naïve state (Lin et al., 2020). *B3GALT5* knockout hESCs show an altered glycosphingolipid profile and a naïve-like transcriptional profile. Finally, *B3galt5* is linked to cerebellar development in a mouse model of cerebellar hypoplasia (Bovio et al., 2019). Disruptor of telomeric silencing 1-like (encoded by *Dot1l*) methylates histone H3, and conditional knockout of *Dot1l* in granule cell precursors causes cerebellar hypoplasia and ataxia. *Dot1l* knockout affects *B3galt5* expression *in vivo*, and inhibition of *Dot1l* in purified granule cell precursors alters H3K79 methylation of the *B3galt5* gene. The authors suggest that *B3galt5* may be a direct target of Dot1l in the developing mouse cerebellum.

Another candidate gene, *ETS2*, is a member of the ETS family of transcription factors, which mediate cell cycle control, proliferation and apoptosis (Sharrocks, 2001). Overexpression of all three human chromosome 21-encoded ETS transcription factors (*ERG*, *ETS2* and *GABPA*) conferred some degree of embryonic lethality in our previous zebrafish screen, and overexpression of *ETS2* inhibited the proliferation of granule cell precursors in the present study (Edie et al., 2018). Overexpression of an *Ets2* cDNA in transgenic mice has been reported to cause craniofacial abnormalities, hypocellularity of the thymus and p53-dependent apoptosis (Sumarsono et al., 1996; Wolvetang et al., 2003b). However, a comparison of thymus and craniofacial phenotypes in Ts65Dn and Ts65Dn, *Ets2^+/+/−^* mice showed that *Ets2* overexpression is not sufficient to produce the phenotypes observed in TgEts2 mice (Hill et al., 2009). To our knowledge, possible cerebellar phenotypes have not been assessed in either TgEts2 or Ts65Dn, *Ets2^+/+/−^* mice. ETS family transcription factors have also been linked to SHH signaling in the developing limb bud, where ETS factors bind to ETS sites in the *Shh* enhancer ZRS to determine the expression pattern of SHH (Lettice et al., 2012). Perhaps most relevant to cerebellar hypoplasia are reports that *ETS2* overexpression induces apoptosis in primary neuronal cultures (Helguera et al., 2005; Wolvetang et al., 2003a). An increase in apoptosis could explain our observation that *ETS2* overexpression inhibits the proliferation of primary granule cell precursors.

The fourth candidate gene to inhibit the proliferation of granule cell precursors is *MIS18A*, which encodes a component of the complex that primes the centromere for recruitment of the centromeric protein A (CENPA) histone following mitosis (Fujita et al., 2007). Knockdown of *MIS18A* in HeLa cells causes chromosome misalignment and missegregation, and *Mis18a^−/−^* mice die early in embryonic development (Fujita et al., 2007; Kim et al., 2012). By contrast, overexpression of MIS18A in U2OS cells increases the amount of CENPA protein present at centromeres (Nardi et al., 2016). It is not obvious how MIS18A overexpression could affect SHH signaling directly. However, cerebellar granule cells constitute more than half of the neurons in the human brain, and granule cell precursors must undergo rapid proliferation to form a population of approximately 50 billion neurons (Llinas et al., 2004). A delay in progression through mitosis or an increase in apoptosis due to overexpression of MIS18A could reduce the number of granule cell precursors available to form the cerebellum.

Our screen focused on inhibitors of SHH activity, but several human chromosome 21 cDNAs consistently activated SHH signaling across cell types. Therapeutic interventions targeting trisomic activators of SHH could worsen SHH-associated phenotypes in people with Down syndrome. In particular, DYRK1A stimulated SHH signaling in our luciferase screens and was previously reported to activate SHH by phosphorylating GLI1 and promoting its retention in the nucleus (Ehe et al., 2017; Mao et al., 2002). Overexpression of *DYRK1A* was previously reported to induce osteoblast differentiation of C3H10T1/2 cells (Mao et al., 2002), although this activation did not reach statistical significance in our screen. DYRK1A is commonly proposed to be a target for treating Down syndrome-associated intellectual disability (Arbones et al., 2019; Atas-Ozcan et al., 2021), but we recommend monitoring potential worsening of phenotypes in SHH-responsive tissues, such as the cerebellum, heart and bone, in preclinical studies of DYRK1A inhibitors (Goodlett et al., 2020; Jamal et al., 2022; Stringer et al., 2017a,b; Thomas et al., 2021).

### Technical limitations of cDNA screens

Our screening paradigm made significant progress towards understanding how the overexpression of human chromosome 21 genes influences SHH signaling. However, no cell culture methods can fully represent the complex effects of trisomy 21 on human development. Overexpression of individual cDNAs cannot reproduce the effects of simultaneous overexpression of more than 500 chromosome 21 genes in the context of trisomy or detect genetic interactions between sets of trisomic genes. Our library contains most conserved chromosome 21 protein-coding genes but does not include several genes that may influence neurodevelopment, including *PCNT* and *SON* (Dingemans et al., 2022; Galati et al., 2018; Kim et al., 2016b; McCurdy et al., 2022). Transient transfection likely causes supraphysiological overexpression of cDNAs, and we did not comprehensively confirm expression of each cDNA, leading to possible false negatives. We also did not compare the extent of overexpression between mRNA injection (Edie et al., 2018), transient transfection and lentiviral transduction, or their concordance with expression levels in human trisomy 21. Overexpression of some cDNAs may cause lethality rather than inhibiting SHH directly; for example, overexpression of the potassium channel subunits *KCNE1* and *KCNJ15* and the mitochondrial subunit *NDUFV3* inhibited SHH but also affected viability in C3H10T1/2 cells. Future work must confirm whether candidate genes modulate SHH *in vivo* and at expression levels mirroring the expected ∼1.5-fold increase observed in trisomy.

The lack of overlap between the four assays presented here and in our previous zebrafish study is also of interest (Edie et al., 2018). The Shh-LIGHT2 and SmoA1-LIGHT assays identify the activation of exogenous transgenes with synthetic *Gli1* promoter in cells with functional canonical SHH pathway signaling, whereas the zebrafish and C3H10T1/2 assays have biological endpoints. The C3H10T1/2 assay shared the greatest number of hits with the zebrafish study, with both assays identifying *JAM2*, *PCBP3* and *RWDD2B*. Overexpression of *LINC00313* (C21orf84) caused U-shaped somites and cyclopia in zebrafish embryos but had no effect in the luciferase and C3H10T1/2 screens. *LINC00313* does not have a known mouse ortholog, but its flanking genes have orthologs on mouse chromosome 17. By conservation of synteny, a yet unidentified mouse ortholog of *LINC00313* is unlikely to account for cerebellar hypoplasia in the Ts65Dn and Ts1Cje models. Because the previous zebrafish screen relied on gross morphological phenotypes rather than a direct readout of SHH signaling, assessing the effects of *B3GALT5*, *ETS2*, *HMGN1* and *MIS18A* overexpression on a zebrafish SHH reporter could help to resolve the discordance between the previous zebrafish and current cellular screens. It is also important to consider the impact of SHH signaling as a morphogen and mitogen in many different cell types throughout life.

### Down syndrome as a complex genetic disorder

Our study established *B3GALT5*, *ETS2*, *HMGN1* and *MIS18A* as likely regulators of proliferation in the developing cerebellum. However, despite completing three parallel screens and a secondary screen, no simplistic answer emerged as to how trisomy 21 causes cerebellar hypoplasia in people with Down syndrome. Past research has devoted itself to identifying ‘the’ chromosome 21 gene responsible for each Down syndrome-associated phenotype. Although studies of individual genes may provide an indication of genes that have major effects for specific phenotypes, they do not deal with complex genetic interactions and compensatory effects. Our findings suggest that for complex developmental phenotypes like intellectual disability, determining the individual effects of trisomic genes across the lifetime may require the development and application of new techniques and the framing of Down syndrome as a complex genetic disorder.

## MATERIALS AND METHODS

### Animals

All procedures met the requirements of the National Institutes of Health Guide for the Care and Use of Laboratory Animals, and were approved by and carried out in compliance with the Johns Hopkins University Animal Care and Use Committee. Founder B6EiC3H-a/A-Ts65Dn (stock no. 001924) (Ts65Dn) mice were obtained from The Jackson Laboratory and maintained as an advanced intercross on a C57BL/6J×C3H/HeJ genetic background. These mice represent the original Davisson strain (Davisson et al., 1990; Moore et al., 2010; Reeves et al., 1995). TcMAC21 mice were generated as previously described and maintained on a C57BL/6J (B6)×DBA/2J (D2) background (Kazuki et al., 2020). TcMAC21 mice are available from The Jackson Laboratory and require an agreement with RIKEN BioResource Research Center and The National University Corporation Tottori University before shipping. Ts65Dn mice were genotyped by PCR and TcMAC21 mice carry a constiutively expressed *GFP* gene on the artificial chromosome and were genotyped by GFP fluorescence using a UV flashlight (NightSea). For RNA-seq, cerebella from pairs of trisomic pups and euploid littermates were isolated from two (Ts65Dn) or three (TcMAC21) litters. Euploid pups for granule cell precursors were C57BL/6J×C3H/HeJ, and cerebella of both sexes were pooled within litters. All experiments were performed on postnatal day 6 (P6).

### Plasmids

Luciferase assays were carried out using the Hsa21 Gene Expression Set in the pCSDest2 vector (https://www.addgene.org/kits/reeves-hsa21-set/). cDNAs for the C3H10T1/2 differentiation assay were subcloned into the pcDNA6.2/EmGFP-Bsd/V5-DEST vector (Invitrogen, V36620) and included full length cDNAs for *KCNE1*, *DOP1B* and *Rcan1*, which were truncated in our original pCSDest2 cDNA library. cDNAs for lentiviral transduction were subcloned into the plenti-CAG-gate-FLAG-IRES-GFP vector (Addgene plasmid #107398; deposited by William Kaelin) (Lu et al., 2014). To facilitate efficient subcloning, the kanamycin resistance gene of the vector was replaced with the ampicillin resistance gene from the pcDNA6.2/EmGFP-Bsd/V5-DEST vector by digesting both vectors with BspHI and ligating with T4 DNA ligase.

Unless otherwise noted, Hsa21 cDNAs were acquired from the Hsa21 Gene Expression Set as previously described and subcloned using Gateway cloning (Edie et al., 2018). *JAM2* cDNA was obtained from the Hsa21 Gene Expression Set and subcloned using TOPO cloning. *KCNE1* (Thermo Fisher Scientific, Ultimate ORF Clone IOH54610) and *TRAPPC10* (Thermo Fisher Scientific, Ultimate ORF Clone IOH53207) in pENTR221 were obtained from Johns Hopkins University Hit Genomics Services. Mouse *Rcan1* in pCMV-SPORT6 (Dharmacon, Mammalian Gene Collection 4236038) was subcloned using TOPO cloning.

The following plasmids were acquired from Addgene: pENTR-DsRed2 N1 (CMB1) (#22523, deposited by Eric Campeau), pDONR223_GLI1_WT (#82123; deposited by Jesse Boehm, William Hahn and David Root; Kim et al., 2016a), pEGFPC3-mSufu [#65431; deposited by Aimin Liu; subcloned using TOPO cloning (Zeng et al., 2010)], pMD2.G (#12259; deposited by from Didier Trono) and psPAX2 (#12260; deposited by Didier Trono). The following plasmids were obtained from The ORFeome Collaboration: *DOP1B* (HsCD00431873) in pENTR223.1 (subcloned using TOPO cloning), *GNAS* (HsCD00288799) in pENTR223 (subcloned using TOPO cloning) and *SHH* (HsCD00082632) in pENTR223.1. *MOSMO* (EX-H4481-M02) in pReceiver-M02 was obtained from GeneCopoeia. Plasmids for transfection were purified using endotoxin-free midiprep kits. Plasmids are available from Addgene (https://www.addgene.org/Roger_Reeves/).

### Cell culture

Mouse Shh-LIGHT2 and SmoA1-LIGHT cells were gifts from Philip Beachy and colleagues and were derived from the original stocks created by this group at Johns Hopkins University (Taipale et al., 2000). Shh-LIGHT2 and SmoA1-LIGHT cells were grown in Dulbecco's Modified Eagle's Medium (DMEM; Gibco, 11965092) supplemented with 10% calf serum (Sigma-Aldrich, C8056 or N4637) and 1% penicillin-streptomycin (Quality Biological, 50-751-7267). Shh-LIGHT2 cultures were kept under antibiotic selection with 400 μg/ml geneticin (Gibco, 10131035) and 150 μg/ml zeocin (Invitrogen, R25001), and SmoA1-LIGHT cells were cultured with 400 μg/ml geneticin and 100 μg/ml hygromycin B (Corning, 30-240-CR). C3H10T1/2 cells [American Type Culture Collection (ATCC), CCL-226] were maintained in DMEM supplemented with 10% fetal bovine serum (HyClone, SH30071.03), 2 mM L-glutamine (Quality Biological, 118-084-721) and 1% penicillin-streptomycin. 293FT cells (Invitrogen, R70007) were maintained in DMEM with 10% fetal bovine serum (HyClone, SH30071.03), 0.1 mM MEM non-essential amino acids (Gibco, 1114050), 6 mM L-glutamine, 1 mM MEM sodium pyruvate (Sigma-Aldrich, S8636) and 1% penicillin-streptomycin with 500 μg/ml geneticin. Primary granule cell precursors were maintained in neurobasal medium (Gibco, 21103049) with 2 mM GlutaMAX (Gibco, 35050061), 1% penicillin-streptomycin, 1 mM sodium pyruvate (Sigma-Aldrich, S8636), 2% B27 (Gibco, 12587010) and 6 nM SAG (Calbiochem, 566661). Cell lines were authenticated as described in the text but were not tested for contamination.

### Luciferase reporter assays

To quantify hedgehog pathway activity in Shh-LIGHT2 cells, the cells were removed from antibiotic-containing medium and seeded in 96-well plates at densities allowing them to reach confluence within 4 days. Two days after seeding, cells were transfected with a plasmid encoding GFP (100 ng/well; two to three rows; 16-24 wells or technical replicates) or one of five Hsa21 genes (100 ng/well; one row, eight wells or technical replicates per unique cDNA) using Lipofectamine 2000 (Invitrogen, 11668030) according to the manufacturer's instructions. On day four, the medium was refreshed with DMEM containing 0.5% calf serum and 100 nM or 1 μM SAG. After 48 h, cells were lysed and Fluc/Rluc activity was quantified using the Dual-Luciferase Reporter Assay System (Promega, E1910) and a 1450 MicroBeta Luminescence Counter (PerkinElmer). For the SmoA1-LIGHT screen, cells were seeded in 96-well plates at densities that would allow them to reach confluence within 2 days. One day after seeding, cells were transfected overnight with plasmids encoding GFP or one of five Hsa21 genes and then switched to DMEM containing 0.5% calf serum for 24 h before quantification of Fluc/Rluc activity.

For both Shh-LIGHT2 and SmoA1-LIGHT screens, the Fluc/Rluc activity was normalized to the median value of the 96-well plate (intra-plate median centering). This process takes into account differences in the absolute intensity values between plates, controls for unintended spatial gradients within plates, such as those that occur along the periphery, and buffers against the presence of signaling outliers. Normalized values were then averaged for each Hsa21 cDNA or control gene. At minimum, all experiments were conducted with sets of eight transfected wells. Technical replicates were averaged and z-scores were calculated for each cDNA (Jacob et al., 2011).

For validation studies of Shh-LIGHT2, cells were cultured to confluency in 96-well plates, then treated with 1 μM SAG, 1 uM fluocinonide (Sigma-Aldrich, SML0099), 1 μM fluticasone (Sigma-Aldrich, F9428) or 10 μM vitamin D3 (Sigma-Aldrich, C9756) in DMEM containing 0.5% calf serum.

### C3H10T1/2 differentiation

To quantify osteoblast differentiation following transfection of Hsa21 cDNAs, approximately 5000 C3H10T1/2 cells were seeded into each well of a 96-well plate. Twenty-four hours later, cells were transfected with 100 ng plasmid DNA per well using Lipofectamine 3000 according to the manufacturer's instructions (Invitrogen, L3000008). The position of each cDNA was randomized between experiments to minimize positional effects. Transfection efficiency was monitored in live cells via GFP expression. Twenty-four hours after transfection, cells were treated with plain medium, 200 nM SAG, 2 μM cyclopamine (Calbiochem, 239806) or 200 nM SAG plus 2 μM cyclopamine. Four days after treatment, cells were washed with PBS and lysed with 50 μl passive lysis buffer (Promega, E194A) for 45 min. To quantify alkaline phosphatase activity, 200 μl alkaline phosphatase blue microwell substrate (Sigma-Aldrich, AB0100) was added to each well, and the plate was incubated in the dark for 30 min. Color development was measured using a SpectraMax 340 Microplate Reader (Molecular Devices) at 600 nm.

cDNAs were screened in two sets for a total of twenty independent replicates per human chromosome 21 cDNA. Alkaline phosphatase activity was normalized to the median value of each plate. Cell viability was assessed 48, 72 and 96 h after transfection using the MTT Cell Proliferation Assay kit (ATCC, 30-1010K) according to manufacturer's instructions.

To stain cells for alkaline phosphatase activity, cells were fixed with 10% neutral buffered formalin (Sigma-Aldrich, HT501320) for 1 min, permeabilized with 0.05% Tween-20 (Sigma-Aldrich, P9416) in PBS, and labeled with BCIP/NBT alkaline phosphatase substrate (Sigma-Aldrich, B5655). Cells were counterstained with Nuclear Fast Red (Amresco, 1B1369) and dehydrated before mounting.

### RNA-seq

RNA-seq was performed as previously described (Kazuki et al., 2020). Briefly, RNA from P6 cerebella was extracted and library preparation was conducted using the NEBNext Poly(A) mRNA Magnetic Isolation Module (E7490, New England Biolabs) and NEBNext Ultra II RNA Library Prep Kit for Illumina (E7770, New England Biolabs). Library quality was assessed with an Agilent 2100 Bioanalyzer. Libraries were sequenced by the Johns Hopkins Single Cell and Transcriptomics Core (NovaSeq SP run, 50 bp paired-end reads) for an average of ∼54 million reads per sample.

Sequencing reads were mapped to the mouse genome mm39 modified by appending human chromosome 21, using the alignment tool STAR v.2.4.2a (Dobin et al., 2013). The aligned reads were assembled with PsiCLASS v.1.0.2 (Song et al., 2019) to create gene and transcript models. Unlike traditional transcript assemblers that process each sample separately, PsiCLASS simultaneously analyzes all samples in the experiment to produce a unified set of transcript annotations to use in the subsequent differential analyses. Transcripts were then assigned to known reference genes from the NCBI RefSeq databases (mouse release October 2020 and human release May 2021) (https://www.gencodegenes.org/mouse/ and https://www.gencodegenes.org/human/). Lastly, DESeq2 (Anders and Huber, 2010) was used to quantify the expression levels and determine the sets of differentially expressed genes. Additional visualizations, including plots of principal coordinate analysis (PCA) components, were visualized with custom R scripts. For comparison of human and mouse orthologs in the TcMAC21 model, trisomic counts were first length normalized using the formula len_norm_readcounts=50× readcounts/genelen, where len_norm_readcounts is the normalized gene expression to be calculated, readcounts is the read count reported for the gene by DESeq2, and genelen is the total length of the exons (over all annotated transcripts) of the gene, after accounting for exon overlaps. Human and mouse counts for each gene were then summed and fold changes were reported as a ratio of TcMAC21 counts to euploid counts. Gene Ontology and gene set enrichment analyses were performed using the R packages gprofiler2 v.0.2.1 (Kolberg et al., 2020), GSVA v.1.42.0 (Hanzelmann et al., 2013), GSEABase v.1.56.0 (https://bioconductor.org/packages/release/bioc/html/GSEABase.html) and clusterProfiler v.4.4.1 (Wu et al., 2021b). Canonical pathways (reactome) gene set for gene set enrichment analysis was retrieved using the msigdbr R package v.7.4.1 (https://igordot.github.io/msigdbr/articles/msigdbr-intro.html). Other R packages used to analyze and visualize RNA-seq data include tidyverse v.1.3.1 (Wickham et al., 2019), cowplot v.1.1.1 (https://wilkelab.org/cowplot/index.html), ggbreak v.0.0.9 (Xu et al., 2021), ggrepel v.0.9.1 (https://github.com/slowkow/ggrepel), RColorBrewer v.1.1-2 (https://CRAN.R-project.org/package=RColorBrewer), gplots v.3.1.3 (https://github.com/talgalili/gplots), and enrichplot v.1.14.2 (https://yulab-smu.top/biomedical-knowledge-mining-book/enrichplot.html) with scripts from DIY.transcriptomics (Berry et al., 2021).

### Lentiviral production

Approximately 750,000 low-passage 293FT cells were seeded into each well of a 6-well plate coated with poly-L-ornithine (Sigma-Aldrich, P2533). One day after seeding, cells were transfected with 640 ng pMD2.G, 975 ng psPAX and 1275 ng lentiviral target plasmid using Lipofectamine 2000 and PLUS reagent (Invitrogen, 11514015). The medium was refreshed 4 h after transfection. The supernatant was collected 48 and 72 h post transfection, filtered with a 0.45 μm filter (Millex-HV, SLHV013SL), and concentrated with Lenti-X Concentrator (Takara Bio, 631231) according to the manufacturer's instructions. Physical titer was determined using the Lenti-X p24 Rapid Titer Kit (Takara Bio, 632200) and granule cell precursors were transduced at an estimated multiplicity of infection of ∼4.

### Granule cell precursor isolation

Cerebella from P6 pups were dissected into ice-cold Hanks' Balanced Salt Solution (HBSS; Gibco, 14170112) with 0.6% glucose, digested with papain (Worthington Papain Dissociation System, LK003150) and triturated with a serum-coated pipette (Lee et al., 2009). Dissociated cells were isolated from membrane fragments on an albumin-ovomucoid inhibitor discontinuous density gradient (Worthington Papain Dissociation System, LK003150). Granule cell precursors were further purified on a 35%/60% Percoll gradient (Sigma-Aldrich, E0414). Viable cells were counted with a Countess II Automated Cell Counter (Thermo Fisher Scientific, A27978), and approximately 100,000 cells were seeded into each well of a 96-well plate coated with poly-L-lysine (Sigma-Aldrich, P4832).

### Granule cell precursor EdU incorporation assay

Twenty-four hours after seeding, granule cell precursors were transduced with lentiviral particles. Infection was monitored via expression of GFP from the IRES-GFP construct. One day after transduction, the medium was refreshed with neurobasal medium containing 6 nM SAG. Two days after transduction, the cells were treated with 15 μM EdU for 24 h. EdU incorporation was quantified using the Click-iT EdU Proliferation Assay for Microplates kit (Invitrogen, C10499) according to the manufacturer's instructions.

### Statistical analysis

Statistical analyses were performed using GraphPad Prism 9.1.2 or R version 4.1.3. For luciferase screens, z-scores were calculated by comparing the Fluc/Rluc ratio for each cDNA to the set of all screened cDNAs. For the C3H10T1/2 alkaline phosphatase screen, non-parametric Kruskal–Wallis test was followed by Dunn's post-hoc test comparing GFP control to all other cDNAs. All other assays were analyzed with two-tailed unpaired Student's *t*-test, one-way ANOVA with Fisher's least significant difference post hoc test, or two-way ANOVA as noted. *P*-values were corrected for multiple comparisons by controlling the false discovery rate using the two-stage linear step-up procedure of Benjamini, Krieger and Yekutieli.

## Supplementary Material

10.1242/dmm.049712_sup1 Supplementary information
