# Supplementary material for: Overexpression screen of chromosome 21 genes reveals modulators of Sonic hedgehog signaling relevant to Down syndrome
Source: Dis Model Mech. 2023 Apr 13;16(4):dmm049712. doi: 10.1242/dmm.049712 (PMC10120076; doi:10.1242/dmm.049712)
Supplement: Supplementary information [file dmm-16-049712-s1.pdf]

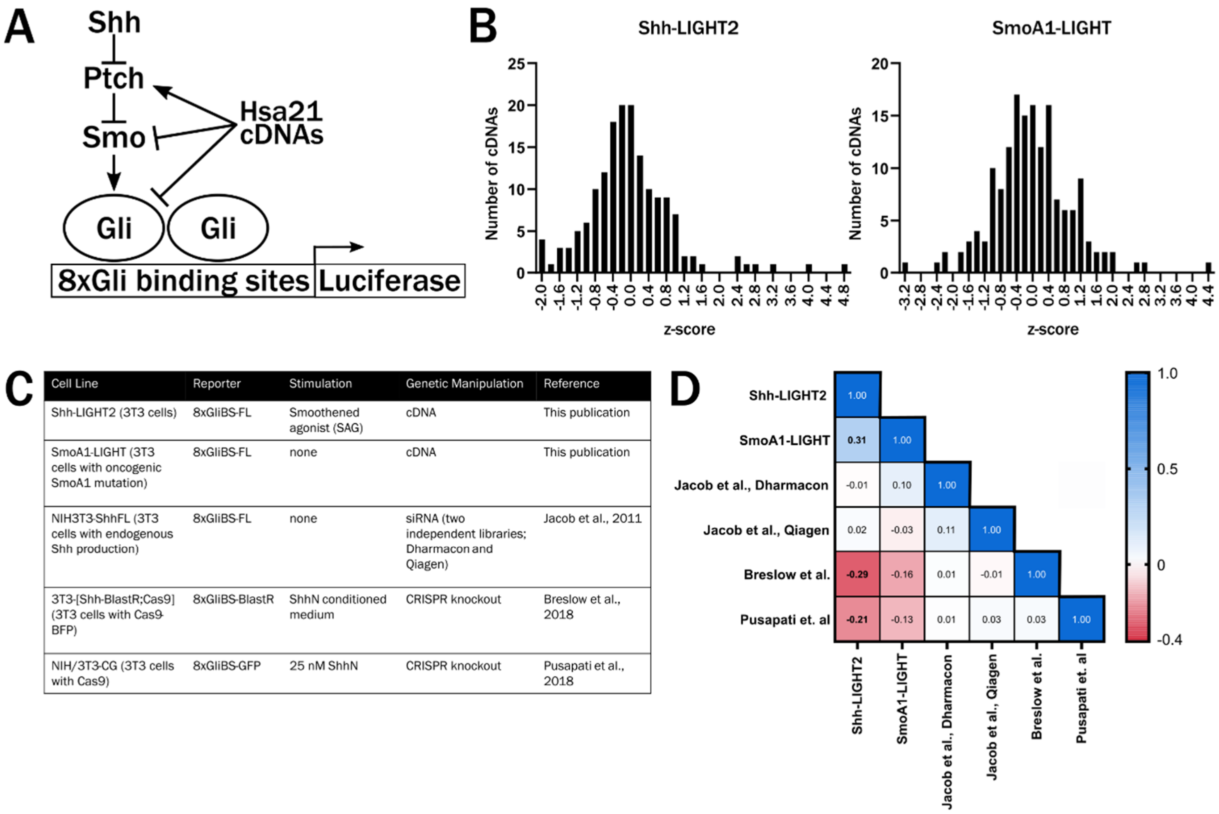

Cell Line	Reporter	Stimulation	Genetic Manipulation	Reference
Shh-LIGHT2 (3T3 cells)	8xGliBS-FL	Smoothened agonist (SAG)	cDNA	This publication
SmoA1-LIGHT (3T3 cells with oncogenic SmoA1 mutation)	8xGliBS-FL	none	cDNA	This publication
NIH3T3-ShhFL (3T3 cells with endogenous Shh production)	8xGliBS-FL	none	siRNA (two independent libraries; Dharmacon and Qiagen)	Jacob et al., 2011
3T3-[Shh-BlastR;Cas9] (3T3 cells with Cas9-BFP)	8xGliBS-BlastR	ShhN conditioned medium	CRISPR knockout	Breslow et al., 2018
NIH/3T3-CG (3T3 cells with Cas9)	8xGliBS-GFP	25 nM ShhN	CRISPR knockout	Pusapati et al., 2018

Fig. S1. Shh-LIGHT2 and SmoA1-LIGHT screens. (A) Schematic of SHH signaling and 8xGliBS-FL reporter. Sonic hedgehog binds to Patched and relieves inhibition of Smoothened, which acts as a transducer to activate signaling via the Gli transcription factors. Binding of Gli to the 8xGliBS-FL promotes transcription of luciferase. Overexpression of chromosome 21 genes may activate or inhibit SHH signaling at any level of the signaling pathway. **(B)** Distribution of z-scores in Shh-LIGHT2 and SmoA1-LIGHT cDNA overexpression screens. **(C)** Summary of previously reported siRNA knockdown and CRISPR knockout screens using the 8xGliBS reporter. Data for individual genes are available in table S6. **(D)** Correlation matrix showing Pearson correlation coefficient r between pairs of screens for the 115 chromosome 21 genes and mouse orthologs with data across all screens. Bolded correlation coefficients have $P < 0.05$. The Shh-LIGHT2 and SmoA1-LIGHT cDNA screens are positively correlated, whereas Shh-LIGHT2 shows a negative correlation with two CRISPR knockout screens.

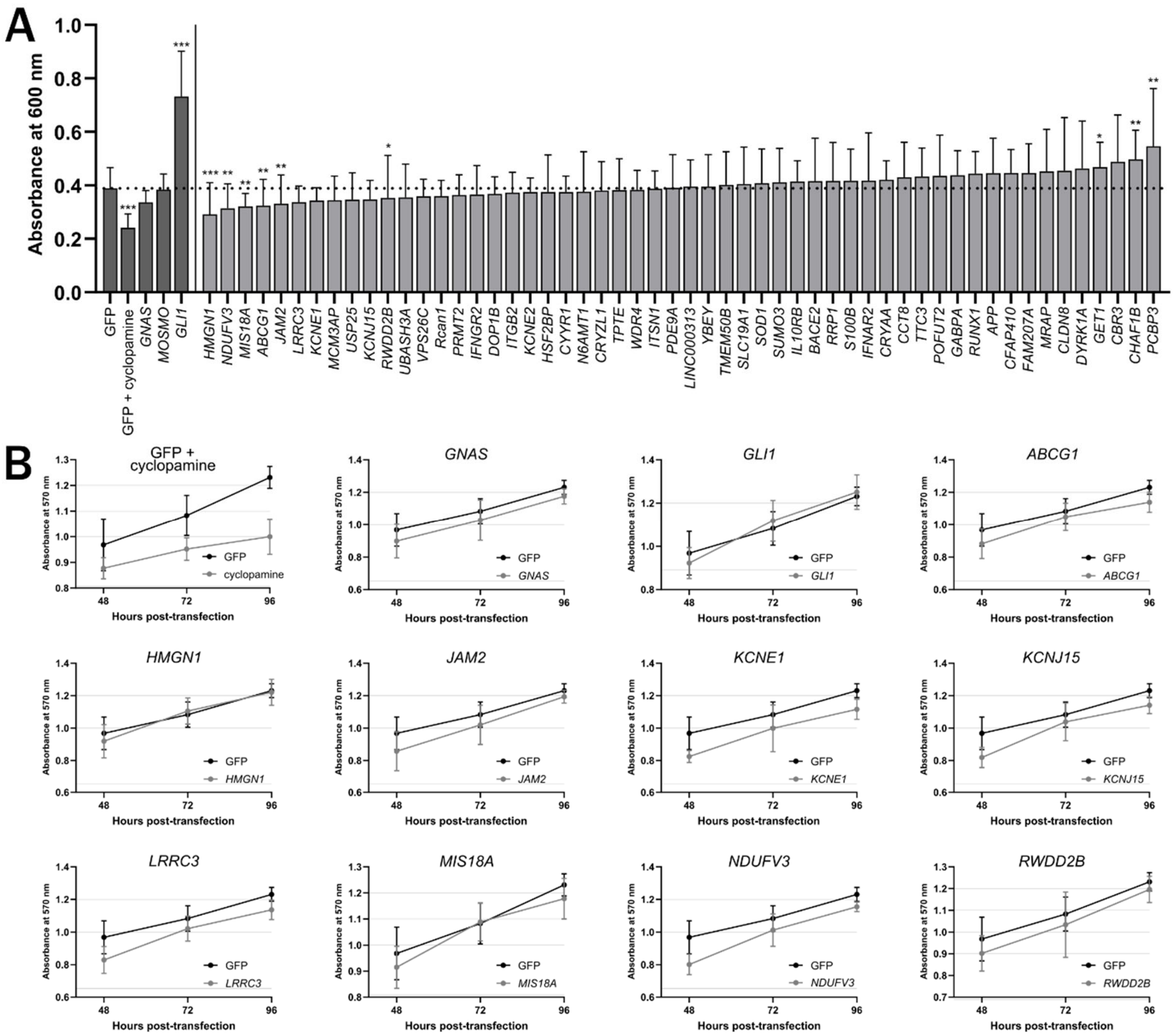

Fig. S2. C3H10T1/2 osteoblast differentiation and viability following transfection of chromosome 21 cDNAs. (A) Quantification of alkaline phosphatase activity following transfection of chromosome 21 cDNAs and stimulation with SAG (n=20). All conditions were compared to GFP control. ***P<0.001, **P<0.01, *P<0.05 (Kruskal-Wallis test followed by Dunn's post-hoc test). **(B)** Quantification of viability of untreated C3H10T1/2 cells 48, 72, and 96 hours post-transfection (n=7). In cells treated with SAG, only cyclopamine treatment affected viability (data not shown).

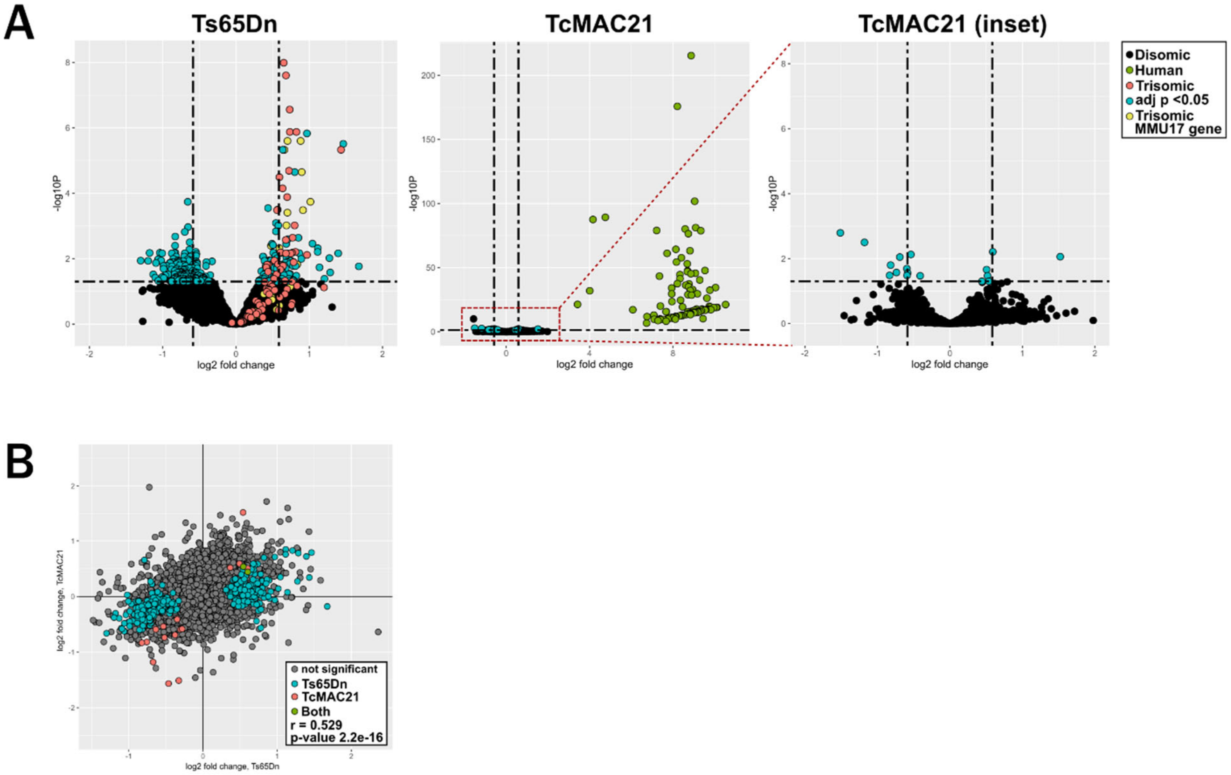

Fig. S3. Expression of disomic genes in Ts65Dn and TcMAC21 cerebellum. (A) Volcano plots showing log2 fold change and -log10(P value) in Ts65Dn and TcMAC21 samples. Teal points represent disomic transcripts with adjusted P <0.05, salmon points represent chromosome 21 orthologs that are trisomic in Ts65Dn, yellow points represent non-chromosome 21 orthologs (MMU17) transcripts that are trisomic in Ts65Dn, and green points represent human transcripts in TcMAC21 samples. **(B)** Scatterplot showing log2 fold change of disomic transcripts in Ts65Dn and TcMAC21 samples. Teal points are significantly differentially expressed in Ts65Dn samples, salmon points are significantly differentially expressed in TcMAC21 samples, and green points are differentially expressed in both Ts65Dn and TcMAC21 samples. Pearson correlation coefficient r=0.529 and P=2.2e-16.

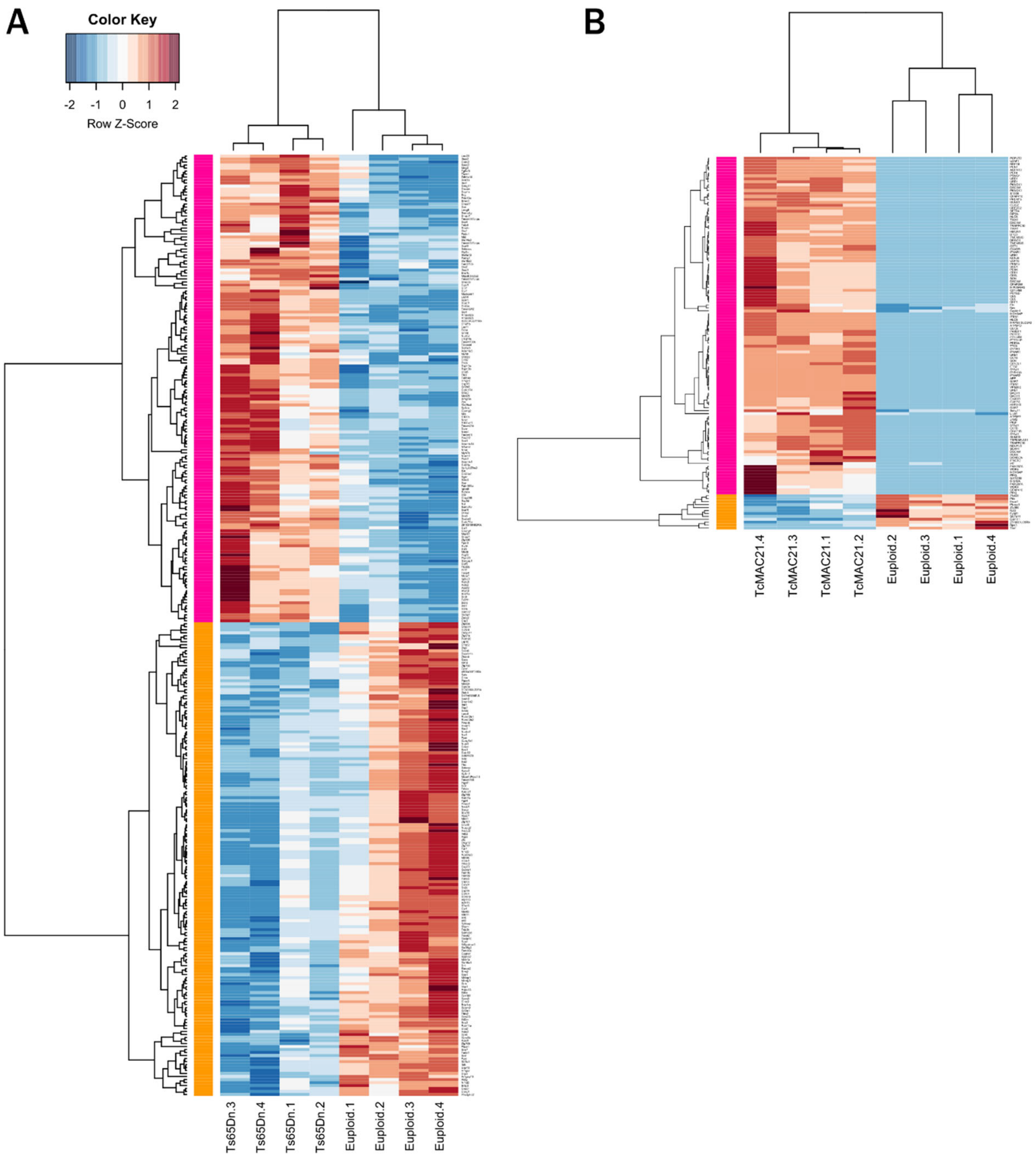

Fig. S4. Unsupervised clustering of differentially expressed genes in Ts65Dn and TcMAC21 samples. (A) Unsupervised clustering of 314 differentially expressed transcripts (rows) in 4 Ts65Dn cerebella and 4 euploid littermates (columns). The orange module represents genes downregulated in Ts65Dn relative to control and the pink module represents genes upregulated in Ts65Dn. (B) Unsupervised clustering of 127 differentially expressed transcripts in 4 TcMAC21 cerebella and 4 euploid littermates. 109/127 differentially expressed transcripts derive from the HSA21q-MAC hybrid chromosome.

Disease Models & Mechanisms • Supplementary information

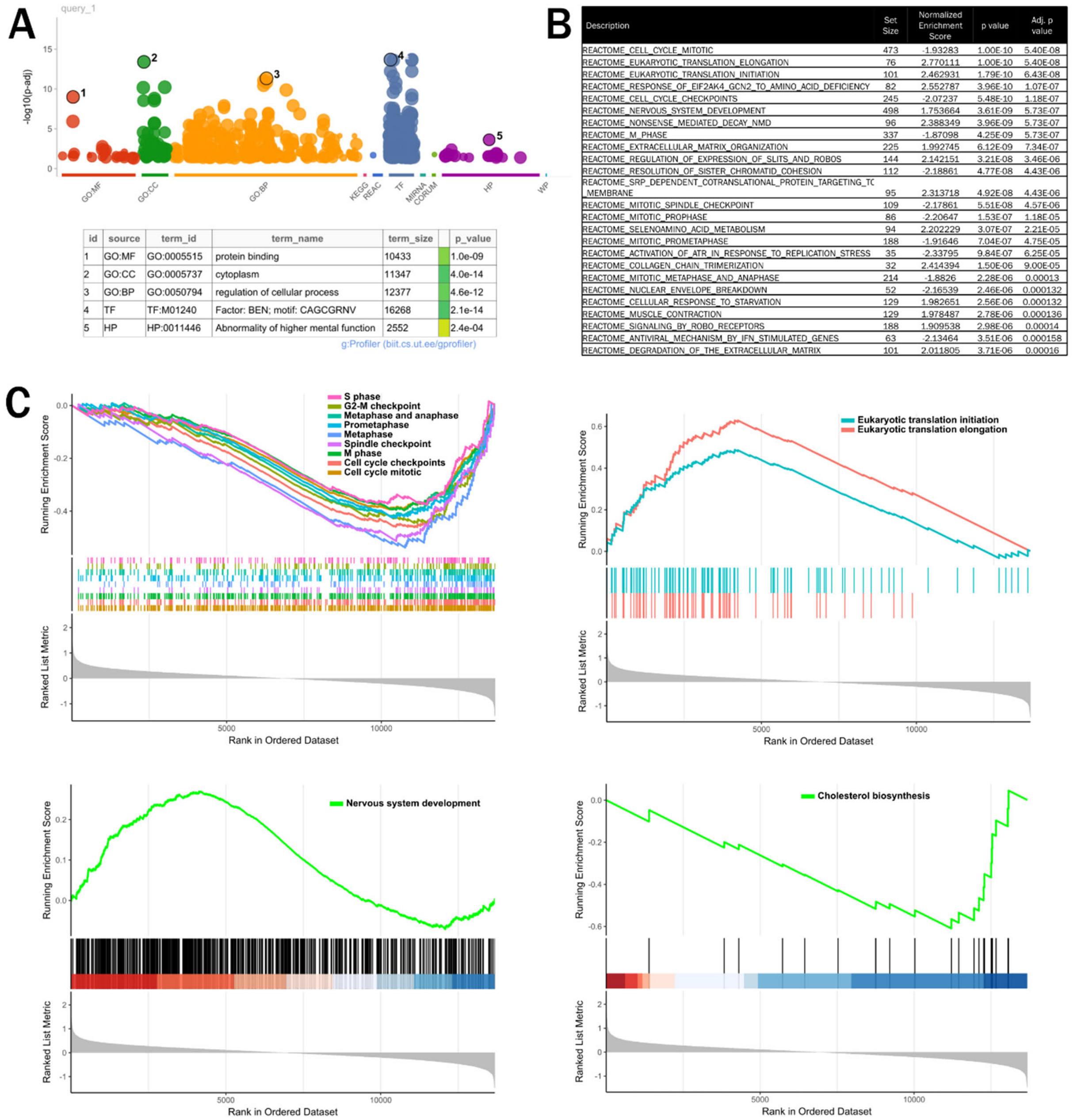

id	source	term_id	term_name	term_size	p_value
1	GO:MF	GO:0005515	protein binding	10433	1.0e-09
2	GO:CC	GO:0005737	cytoplasm	11347	4.0e-14
3	GO:BP	GO:0050794	regulation of cellular process	12377	4.6e-12
4	TF	TF:M01240	Factor: BEN; motif: CAGCGRNV	16268	2.1e-14
5	HP	HP:0011446	Abnormality of higher mental function	2552	2.4e-04

Description	Set Size	Normalized Enrichment Score	p value	Adj. p value
REACTOME_CELL_CYCLE_MITOTIC	473	-1.93283	1.00E-10	5.40E-08
REACTOME_EUKARYOTIC_TRANSLATION_ELONGATION	76	2.770111	1.00E-10	5.40E-08
REACTOME_EUKARYOTIC_TRANSLATION_INITIATION	101	2.462931	1.79E-10	6.43E-08
REACTOME_RESPONSE_OF_EIF2AK4_GCN2_TO_AMINO_ACID_DEFICIENCY	82	2.552787	3.96E-10	1.07E-07
REACTOME_CELL_CYCLE_CHECKPOINTS	245	-2.07237	5.48E-10	1.18E-07
REACTOME_NERVOUS_SYSTEM_DEVELOPMENT	498	1.753664	3.61E-09	5.73E-07
REACTOME_NONSENSE_MEDIATED_DECAY_NMD	96	2.388349	3.96E-09	5.73E-07
REACTOME_M_PHASE	337	-1.87098	4.25E-09	5.73E-07
REACTOME_EXTRACELLULAR_MATRIX_ORGANIZATION	225	1.992745	6.12E-09	7.34E-07
REACTOME_REGULATION_OF_EXPRESSION_OF_SLITS_AND_ROBOS	144	2.142151	3.21E-08	3.46E-06
REACTOME_RESOLUTION_OF_SISTER_CHROMATID_COHESION	112	-2.18861	4.77E-08	4.43E-06
REACTOME_SRP_DEPENDENT_COTRANSLATIONAL_PROTEIN_TARGETING_TO_MEMBRANE	95	2.313718	4.92E-08	4.43E-06
REACTOME_MITOTIC_SPINDLE_CHECKPOINT	109	-2.17861	5.51E-08	4.57E-06
REACTOME_MITOTIC_PROPHASE	86	-2.20647	1.53E-07	1.18E-05
REACTOME_SELENOAMINO_ACID_METABOLISM	94	2.202229	3.07E-07	2.21E-05
REACTOME_MITOTIC_PROMETAPHASE	188	-1.91646	7.04E-07	4.75E-05
REACTOME_ACTIVATION_OF_ATR_IN_RESPONSE_TO_REPLICATION_STRESS	35	-2.33795	9.84E-07	6.25E-05
REACTOME_COLLAGEN_CHAIN_TRIMERIZATION	32	2.414394	1.50E-06	9.00E-05
REACTOME_MITOTIC_METAPHASE_AND_ANAPHASE	214	-1.8826	2.28E-06	0.00013
REACTOME_NUCLEAR_ENVELOPE_BREAKDOWN	52	-2.16539	2.46E-06	0.000132
REACTOME_CELLULAR_RESPONSE_TO_STARVATION	129	1.982651	2.56E-06	0.000132
REACTOME_MUSCLE_CONTRACTION	129	1.978487	2.78E-06	0.000136
REACTOME_SIGNALING_BY_ROBO_RECEPTORS	188	1.909538	2.98E-06	0.00014
REACTOME_ANTIVIRAL_MECHANISM_BY_IFN_STIMULATED_GENES	63	-2.13464	3.51E-06	0.000158
REACTOME_DEGRADATION_OF_THE_EXTRACELLULAR_MATRIX	101	2.011805	3.71E-06	0.00016

Fig. S5. Gene ontology and gene set enrichment analyses of differentially expressed genes in Ts65Dn cerebellum. (A) Manhattan plot showing top gene ontology terms identified in Ts65Dn cerebellum. Differentially expressed genes contributing to "abnormality of higher mental function" are listed in figure S6. **(B)** Top 25 pathways identified by gene set enrichment analysis in Ts65Dn samples. **(C)** Gene set enrichment analysis for pathways significantly enriched in Ts65Dn samples (translation and nervous system development) and pathways enriched in control samples (mitotic/cell cycle and cholesterol biosynthesis).

Disease Models & Mechanisms • Supplementary information

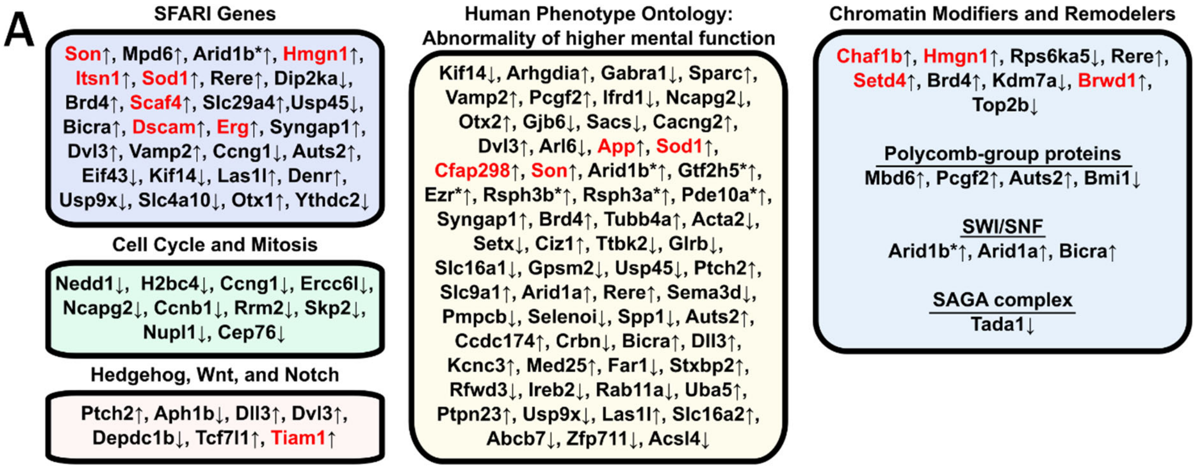

Fig. S6. Differentially expressed genes in Ts65Dn cerebellum are implicated in human neurodevelopmental disorders, mitosis, and chromatin remodeling. (A) Subset of differentially expressed genes ($q < 0.05$) identified in Ts65Dn grouped by cellular and disease processes. Up arrows signify genes that are upregulated in Ts65Dn samples, asterisks signify genes that are trisomic in Ts65Dn mice but are not orthologs of chromosome 21 genes, and red signifies trisomic genes that are orthologs of chromosome 21 genes. Differentially expressed genes include 28 in the SFARI Gene database of autism susceptibility loci and others related to cell cycle/mitosis, key developmental pathways including SHH, and chromatin modifiers and remodelers.

Disease Models & Mechanisms • Supplementary information

Table S1. Cerebellar Volumes of Down Syndrome Models

Click here to download Table S1

Table S2. Manual Annotation of HSA21 Genes

Click here to download Table S2

Table S3. Plasmid Information

Click here to download Table S3

Table S4. Shh-LIGHT2 Screen

Click here to download Table S4

Table S5. SmoA1-LIGHT Screen

Click here to download Table S5

Table S6. Screen Comparisons

Click here to download Table S6

Table S7. Ts65Dn RNA-seq in P6 Cerebellum

Click here to download Table S7

Table S8. TcMAC21 RNA-seq in P6 Cerebellum

Click here to download Table S8

Table S9. Gene Set Enrichment Analysis in Ts65Dn Cerebellum

Click here to download Table S9

Table S10. Summary of Expression Data in Human and Mouse Cerebellum

Click here to download Table S10
